# Fluorescent Phthalocyanine-Encapsulated Bovine Serum Albumin Nanoparticles: Their Deployment as Therapeutic Agents in the NIR Region

**DOI:** 10.3390/molecules26154679

**Published:** 2021-08-02

**Authors:** Raluca Borlan, Daria Stoia, Luiza Gaina, Andreea Campu, Gabriel Marc, Maria Perde-Schrepler, Mihaela Silion, Dana Maniu, Monica Focsan, Simion Astilean

**Affiliations:** 1 Biomolecular Physics Department, Faculty of Physics, Babes-Bolyai University, 400084 Cluj-Napoca, Romania; raluca.borlan@ubbcluj.ro (R.B.); dana.maniu@ubbcluj.ro (D.M.); 2 Nanobiophotonics and Laser Microspectroscopy Centre, Interdisciplinary Research Institute on Bio-Nano-Sciences, Babes-Bolyai University, 400271 Cluj-Napoca, Romania; daria.stoia@stud.ubbcluj.ro (D.S.); andreea.campu@ubbcluj.ro (A.C.); 3 The Research Centre on Fundamental and Applied Heterochemistry, Department of Chemistry, Faculty of Chemistry and Chemical Engineering, Babes-Bolyai University, 400028 Cluj-Napoca, Romania; ioana.gaina@ubbcluj.ro; 4 Department of Pharmaceutical Chemistry, Faculty of Pharmacy, ‘Iuliu Hațieganu’ University of Medicine and Pharmacy, 41 Victor Babes Street, 400012 Cluj-Napoca, Romania; marc.gabriel@umfcluj.ro; 5 Department of Radiobiology and Tumor Biology, Oncology Institute Prof. Dr. Ion Chiricuta, 400015 Cluj-Napoca, Romania; pmariaida@yahoo.com; 6 Physics of Polymers and Polymeric Materials, “Petru Poni” Institute of Macromolecular Chemistry, Romanian Academy, 700487 Iasi, Romania; silion.mihaela@icmpp.ro

**Keywords:** NIR photoactive molecules, albumin-based nanoparticles, dual phototherapy, NIR fluorescent agents, ovarian cancer

## Abstract

In recent times, researchers have aimed for new strategies to combat cancer by the implementation of nanotechnologies in biomedical applications. This work focuses on developing protein-based nanoparticles loaded with a newly synthesized NIR emitting and absorbing phthalocyanine dye, with photodynamic and photothermal properties. More precisely, we synthesized highly reproducible bovine serum albumin-based nanoparticles (75% particle yield) through a two-step protocol and successfully encapsulated the NIR active photosensitizer agent, achieving a good loading efficiency of 91%. Making use of molecular docking simulations, we confirm that the NIR photosensitizer is well protected within the nanoparticles, docked in site I of the albumin molecule. Encouraging results were obtained for our nanoparticles towards biomedical use, thanks to their negatively charged surface (−13.6 ± 0.5 mV) and hydrodynamic diameter (25.06 ± 0.62 nm), favorable for benefitting from the enhanced permeability and retention effect; moreover, the MTT viability assay upholds the good biocompatibility of our NIR active nanoparticles. Finally, upon irradiation with an NIR 785 nm laser, the dual phototherapeutic effect of our NIR fluorescent nanoparticles was highlighted by their excellent light-to-heat conversion performance (photothermal conversion efficiency 20%) and good photothermal and size stability, supporting their further implementation as fluorescent therapeutic agents in biomedical applications.

## 1. Introduction

After heart disease, cancer is the second most frequent cause of death around the world, with over 4.5 million new cases and over 2 million deaths in 2018 [1]. Therefore, in the past decades, scientists have searched for new ways to fight against cancer, and the past years have brought huge progress in the field of nanotechnology, especially in the biomedical area. At present, researchers make use of nanotechnology in medical applications to develop nanoparticles (NPs) able to deliver contrast agents and other substances of interest to tumor sites and, at the same time, tailored to fight cancer through different types of therapy [2,3]. For example, gold NPs have been developed together with indocyanine green in order to achieve a hybrid nano-system to perform both photodynamic and photothermal therapy for an innovative targeted and efficient therapeutic strategy [4].

An up-and-coming cancer treatment method is photodynamic therapy (PDT). For this therapy to work, three elements, namely a photosensitizer (PS) agent, PS specific excitation wavelength light, and intracellular oxygen, are indispensable. If we combine these three elements, they produce photochemical reactions that lead to the generation of cytotoxic reactive oxygen species, predominantly singlet oxygen (^1^O_2_). This reactive oxygen species can trigger the patient’s immune system, can close the vasculature around the tumor, and, most importantly, can cause immediate cancer cell death [5,6]. Champeau M. et al. explained that, in the dermatological field, PDT is implemented as a treatment for skin cancer; more specifically, most clinical trials use 5-aminolevulinic acid in high doses as a cream to cover the lesions [6]. After a predefined incubation period, the lesion is exposed to a laser, usually of 635 nm, to stimulate the PS agent. After the PS molecule is stimulated, PDT occurs.

Another cancer therapy that has potential to be translated to clinical use is photothermal therapy (PTT). This technique also implies the use of electromagnetic radiation, and, at present, the most used spectral domain for this type of therapy is the visible one. Aside from light, this therapy involves thermal agents that absorb and then convert light into heat when the electromagnetic radiation has a suiting wavelength. In biomedical applications, these thermal treatments rely on the local heating of the tissue or desired organ in a very specific time frame. In humans, temperature plays a very important role; thus, when the body temperature exceeds 37 °C, it is a sign of a cold, an infection, or another condition that could put our health in danger [7]. If the temperature rises above 42 °C [7], the cells from the targeted tissue will undergo necrosis, and if it reaches 60 °C [7], protein denaturation takes place instantly and is permanent. This kind of treatment is very efficient in theory, but, in real life, it presents significant drawbacks when using free thermal agents due to their lack of distinction between healthy and cancerous tissues [7,8,9]. Rajkumar et al. performed PTT combined with chemotherapy on cervical cancer cells, namely the HeLa cellular line, through loaded NPs with fluorescein isothiocyanate and doxorubicin [10]. For a more specific targeting of these cancer cells, the NPs were additionally functionalized with folic acid. After incubating HeLa cells with the NPs, the samples were irradiated for 5 min with a 532 nm laser that increased their temperature by 13 °C.

Despite the restraints encountered when exciting biological samples with light from the visible spectrum, such as low level of tissue penetration, currently, most medical applications use therapeutic and contrast agents that possess absorption and emission properties in the visible domain [11]. Therefore, to combat their drawbacks, in the past years, researchers have developed and implemented nano-sized agents in pre-clinical studies and clinical trials that are able to encapsulate photoactive molecules in the NIR domain. Unlike the ultraviolet and visible spectrum, NIR electromagnetic radiation penetrates the biological tissue deeper (500 μm up to 1–2 cm) because fluids and biological tissue present a minimal absorption between 650–1400 nm, hereby acquiring the name *biological transparency window* [12]. Anna Oniszczuk et al. [13] claims that the most appropriate irradiation for the human body is with light that has a wavelength of 630 nm or above. However, despite the fact that the Food and Drug Administration has approved many fluorophores for medical use with emission properties in the ultraviolet and visible region of the electromagnetic spectrum, only two emit light in the NIR domain (650–900 nm), namely indocyanine green and methylene blue [4,14].

In recent years, dyes from the phthalocyanine family gained attention in biomedical research as contrast and therapeutic NIR agents. These synthetic dyes are made out of four isoindole units interconnected with nitrogen atoms, forming a large ring of 18 π electrons, and were firstly synthesized in 1928 out of pure accident, but their efficacy as dyes and toners was established only in 1934. Phthalocyanines are usually used in various fields, e.g., chemical sensors, intrinsic semiconductors, etc. and are thoroughly studied as photosensitive chemical compounds for PDT, thanks to their high photostability and good chemical and thermal stability. However, the use of free phthalocyanines in biological applications is limited because they are insoluble in water and therefore tend to create aggregates [15,16,17].

Based on the assumption that a functional dye must fulfil a series of requirements to be simultaneously a good NIR PS in PDT [18] and a fluorescent agent for NIR bio-imaging [19], for this study, the phthalocyanine derivatives were considered, being known as light-sensitive molecules having the absorption and emission maxima in the red/near IR region and a high level of absorption molar coefficient, respectively. The metal ion coordinated in the central cavity of the phthalocyanine plays an important role in the fluorescence emission, singlet oxygen generation, intersystem-crossing efficacy, energy and lifetime of the triplet state [20], and in solution aggregation behavior [21]. As shown by Tasso et al., the Zn(II) ion from zinc(II)phthalocyanine is one of the most efficient _1_O^2^ producers and is able to enhance the fluorescence emission, and the intermolecular aggregation can be more easily disrupted in comparison with metalophthalocyanine containing Cu, Ni, Co, or Pd ions [20].

One promising product of nanotechnological advancements in therapeutic applications, detection, and visualization of cancerous tissues is NPs. They can be used as carriers for different PS, thermal, and contrast agents throughout the human body to cancerous cells, in order to eliminate all unwanted drawbacks and side effects of dispensing free agents, such as poor biocompatibility, low photostability, and non-specific targeting. Hereby, special consideration was given to protein NPs, being biodegradable, being biocompatible, and having a versatile chemical structure [22,23], especially albumin NPs. Albumin is the most abundant protein in plasma, able to encapsulate a wide range of dyes, drugs, PS agents, thermal agents, and other biologically active compounds [23,24], and, for commercial purposes, it can be derived from egg white, rat serum, bovine serum (BSA), and human serum. BSA’s chemical structure is formed of 583 amino acids, having a molecular weight of 69.3 kDa, and, in the past years, NPs synthesized with this protein have been heavily tested in many preclinical studies [25,26]. Moreover, the dimension of BSA NPs can be controlled so that they are in the optimum domain to seize the enhanced permeability and retention effect (EPR).

In the current clinical context, where significant effort is invested in the development of new approaches for relevant medical applications, the purpose of this study is to design state-of-the-art contrast and therapeutic agents with optical features in the NIR region of the electromagnetic spectrum. The newly synthesized NIR fluorescent photoactive molecule, namely Zinc(II)-2,9,16,23-tetraamiophthalocyanine (hereinafter denoted as phthaloNH_2_), able to synergistically combine photothermal and photodynamic therapeutic effects, has been selected for this study. Furthermore, for improved performance and a safe biomedical administration, we encapsulated, in a controlled manner, the NIR fluorophore within protein NPs. Using an adapted fabrication strategy [27], the phthaloNH_2_-loaded protein NPs were designed to meet the following requirements: they (i) present a high photoactive molecule loading capacity, (ii) present reproduction in synthesis, (iii) perform good therapeutic effects, (iv) are biocompatible, etc. PhthaloNH_2_ is a dye in the family of phthalocyanines, able to act at the same time as a PS, and as thermal and contrast agent; the fine equilibrium between the three functions of the NIR molecule allows us to implement phthaloNH_2_ as a multimodal therapeutic and contrast agent. The fluorescence capacity and in vitro biocompatibility of the phthaloNH_2_ loaded BSA NPs (hereinafter denoted as BSA&phthaloNH_2_ NPs) were successfully tested, foreseeing promising outcomes in future studies as an NIR contrast agent in the imaging of living cells. Moreover, even though the relation between the emissive properties, light-to-heat conversion, and ^1^O_2_ generation yields of the photoactive molecules changed once encapsulated within the BSA NPs, their potential to operate as dual therapeutic agents through the synergistic integration of photodynamic and photothermal properties on a single therapeutic agent was evaluated, in this way creating a highly efficient nano-sized phototheranostic agent for cancer treatment.

## 2. Results

### 2.1. Optical Characterization of PhthaloNH_2_

Phthalocyanine synthesis implies a macrocyclization reaction, using starting materials which fall in two different categories: the first one which needs an extra source of nitrogen (phthalic acids, phthalic anhydrides, phthalimide) and the second one which does not necessarily need an external nitrogen source (phthalonitriles, isoindoline-1,3-diimine, 2-cyanobenzamide, phthalimide) and a metal halide or salt for metalated phthalocyanine, respectively. The best alternative for the preparation of Zn(II) tetraamine phthalocyanine [28,29] is the reduction of the nitro group of Zn (II)tetranitro phthalocyanine in the presence of disodium sulfide. The nitro precursor can be synthesized by tetracyclization of nitrophthalonitrile [30] or nitrophthalamide [28].

The newly synthesized phthaloNH_2_ dye was obtained, briefly, from mixing Zinc(II) tetranitrophthalocyanine and Na_2_S·9H_2_O. After purification, the dye was further encapsulated in BSA-based NPs following a two-step procedure: (i) desolvation and (ii) cross-linking.

**Figure 1:**
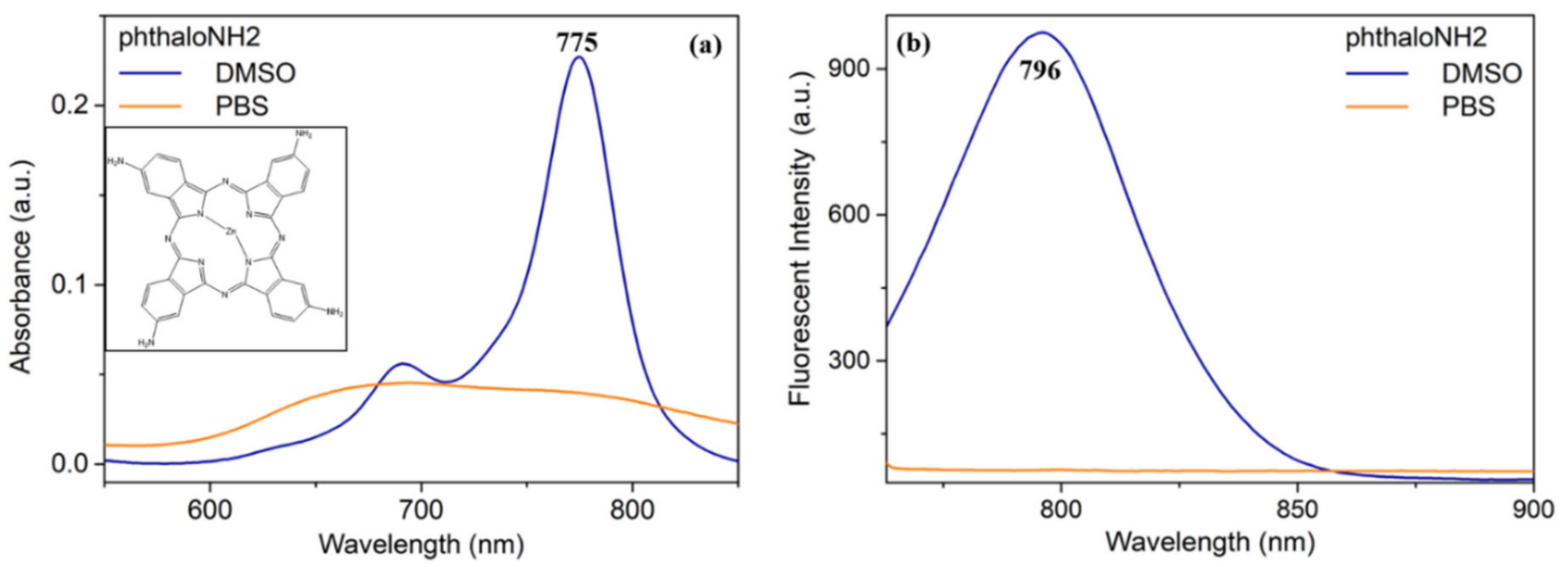
Absorbance (**a**) and fluorescence emission (**b**) spectra of the free phthaloNH_2_ molecules in DMSO (blue) and PBS (orange) solutions. The chemical structure of phthaloNH_2_ can be found in inset (**a**). For both samples, the excitation wavelength was fixed at 745 nm. We used a concentration of 10^−5^ M phthaloNH_2_.

First, using UV-Vis-NIR absorbance (Figure 1a) and fluorescence spectroscopy (Figure 1b), we were able to characterize the photoactive agent in different solvent solutions, PBS and DMSO. Considering that the used photoactive agent is actually a predominantly hydrophobic molecule, we can easily observe from Figure 1 that phthaloNH_2_ presents both absorbance and fluorescence properties only when dissolved in DMSO solution (blue spectra), showing an absorption maximum at 775 nm and an emission maximum at 796 nm. On the contrary, if dissolved in PBS solution (orange spectra), the absorbance spectrum aspect of the phthaloNH_2_ is strongly modified, and the fluorescence emission is lost. Therefore, we can conclude that to preserve phthaloNH_2_s properties, we need to dissolve it only in aprotic polar solvents, specifically the highly polar DMSO. Thus, for the photoactive molecule to maintain its phototheranostic properties under physiological conditions, and for a safe in vivo administration, the encapsulation of the NIR dye within biocompatible nano-carriers is recommended, i.e., protein-based NPs.

### 2.2. PhthaloNH_2_ Loaded BSA NPs

#### 2.2.1. Spectroscopic and Morphologic Characterization

**Figure 2:**
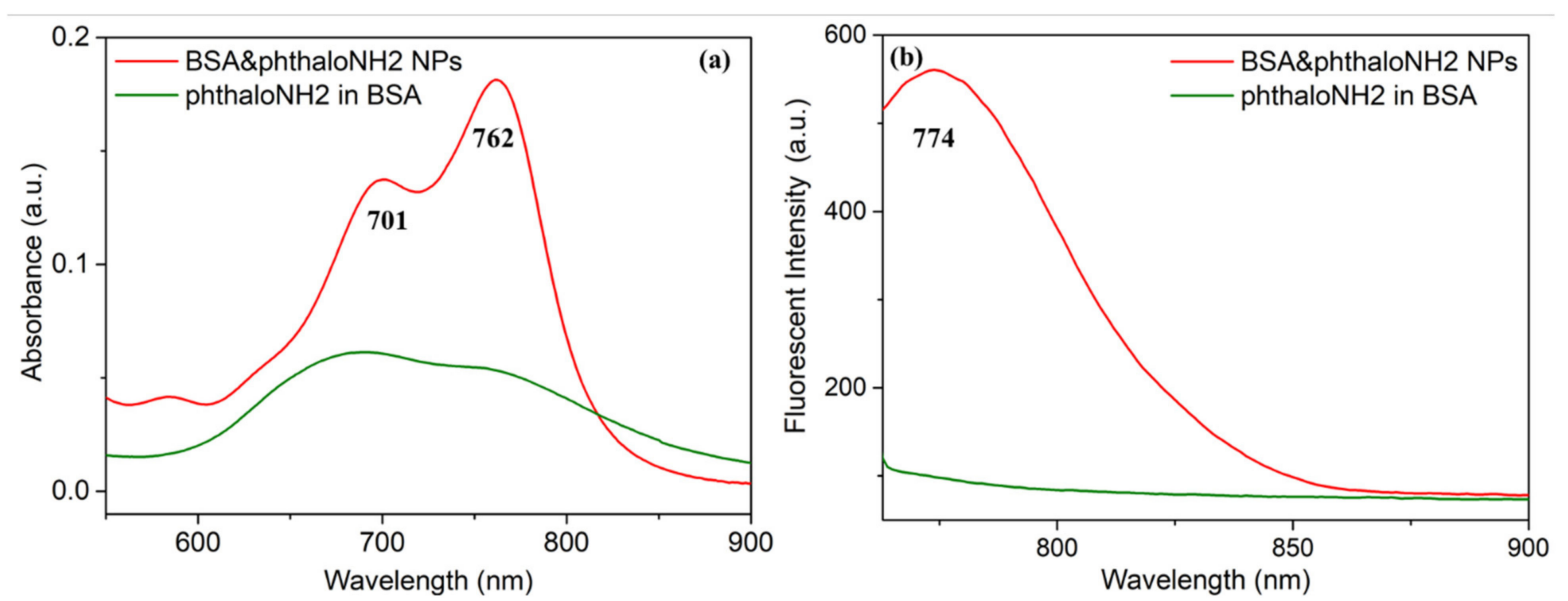
Absorbance (**a**) and fluorescence emission (**b**) spectra of the BSA&phthaloNH_2_ NPs (red) and phthaloNH_2_ in BSA solution (green). For both samples, the excitation wavelength was fixed at 745 nm. We used a concentration of 10^−5^ M phthaloNH_2_.

As depicted in Figure 2a, the phthaloNH_2_ dispersed in BSA aqueous solution (green spectra) presents a broad absorption spectrum in the region of the NIR molecule, implying its mild degradation, while no meaningful fluorescence emission was noticed (Figure 2b). However, the BSA&phthaloNH_2_ NPs present, by comparison, a strong fluorescence intensity and a characteristic absorption spectrum (Figure 2a red spectrum), with a maximum at 762 nm and an emission maximum at 774 nm, confirming the formation of the BSA NPs and protection of the phthaloNH_2_ molecules within them. In accordance with Diac et al., the calculated relative fluorescence quantum yield of the newly synthesized BSA&phthaloNH2 NPs is ϕ = 17% [31]. As standard reference, we used the fluorescence quantum yield of indocyanine green [32]. The second smaller maximum (701 nm) observed in Figure 2a can be assigned to a weak transition to the first excited state (S_0_ → S_1_), namely Q bands, attributed to the π-π* transitions [33].

**Figure 3:**
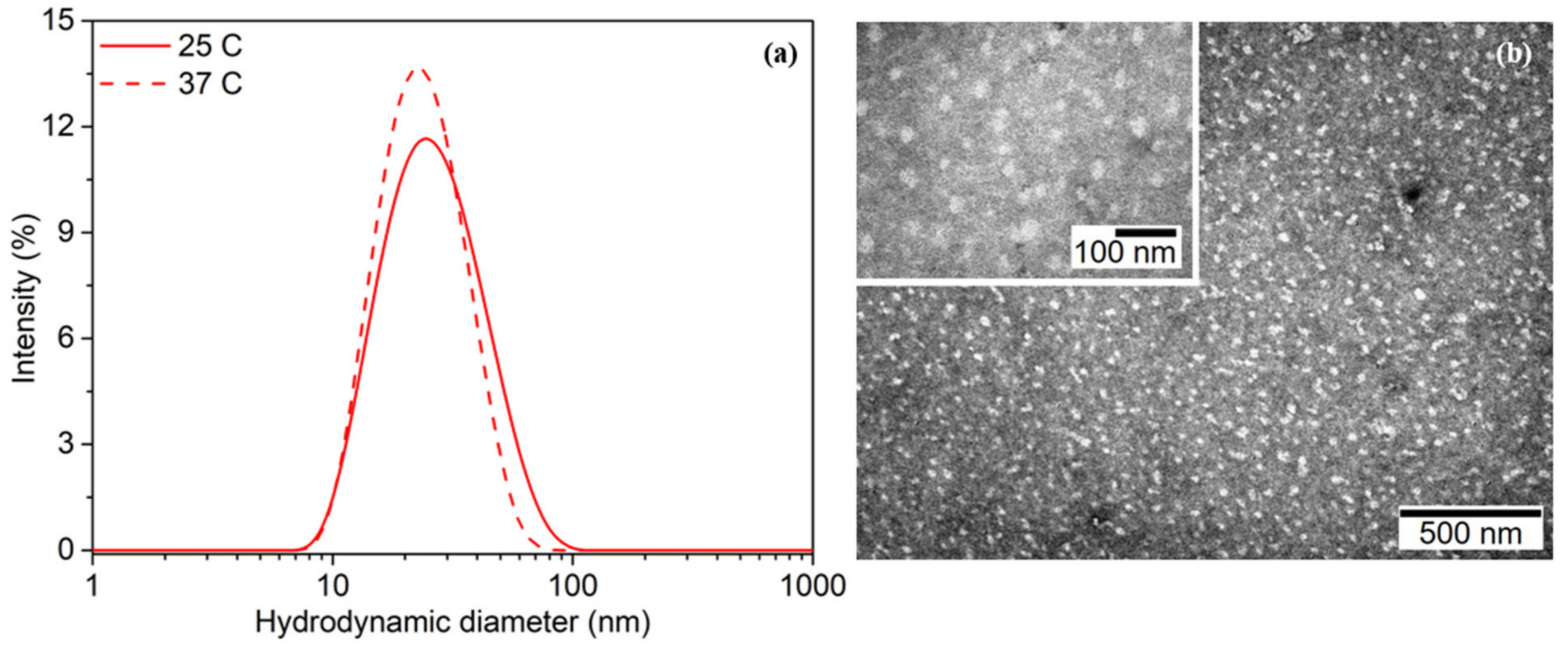
(**a**) DLS spectra of the BSA&phthaloNH_2_ NPs at 25 °C (solid line) and at 37 °C (dashed line); (**b**) TEM images of the afresh synthesized BSA&phthaloNH_2_ NPs.

DLS and Zeta potential measurements of the recorded BSA&phthaloNH_2_ NPs, both at 25 °C and at 37 °C, suggest the promising potential of these protein-based NPs for translation to biomedical use, because they present a negatively charged surface (−12.6 ± 0.9 mV at 25 °C and −13.6 ± 0.5 mV at 37 °C) and a hydrodynamic diameter (Figure 3a) of 28.54 ± 0.42 nm and PDI 0.194 ± 0.027 at 25 °C (solid line) and 25.06 ± 0.62 nm and PDI 0.143 ± 0.008 at 37 °C (dashed line). The quite similar zeta potential (Figure S1 in Supplementary Materials) and diameter measured at 25 °C and 37 °C, respectively, underline the good stability and high potential of the BSA&phthaloNH_2_ NPs to be translated in biomedical applications, these values also being in the optimal range to benefit from the EPR effect. The TEM investigations presented in Figure 3b confirmed the size of the BSA NPs.

Moreover, even after 3 months of storage, the hydrodynamic diameter of the NPs is stable, as proven by DLS measurements (Figure S2a), while 71.4% of the initial fluorescence intensity is maintained (Figure S2b). The aspect of the BSA&phthaloNH_2_ NPs at the initial time and 3 months after the synthesis (Figure S2c) remains consistent, thus being additional proof that the BSA&phthaloNH_2_ NPs are stable upon storage. It is worth mentioning that the NPs were kept in storage at 4 °C and protected from light.

#### 2.2.2. BSA NPs Yield and phthaloNH_2_ Loading Efficiency

Making use of the calibration curve (Figure S3a) calculated for different concentrations of BSA solutions, we determined the BSA&phthaloNH_2_ NPs particle yield to be 75.8 ± 1.3% by employing Equation (1). For absorbance measurements, the optical density of all samples was recorded at 277 nm. These results confirm the great reproductivity of our afresh designed BSA&phthaloNH_2_ NPs, together with the tremendous formation rate of the particles. The loading efficiency of phthaloNH_2_ was calculated using the previously established phthaloNH_2_ calibration curve (Figure S3b) and Equation (2); the optical density of all samples was recorded at 774 nm. The obtained value, 91.14 ± 1.47%, affirms the high amount of phthaloNH_2_ confined within the BSA NPs and, undoubtedly, the excellent loading efficiency of our BSA&phthaloNH_2_ NPs. Standard errors were calculated by triplicate analyses.

(1) $$\mathrm{Particle}\;\mathrm{yield}\;(\%)=\frac{\mathrm{BSA}\;\mathrm{final}\;\mathrm{quantity}}{\mathrm{BSA}\;\mathrm{initial}\;\mathrm{quantity}}\times 100$$

(2) $$\mathrm{Loading}\;\mathrm{efficiency}\;(\%)=\frac{{\mathrm{Initial}\;\mathrm{amount}\;\mathrm{of}\;\mathrm{phthaloNH}}_{2}-{\mathrm{Amount}\;\mathrm{of}\;\mathrm{phthaloNH}}_{2}\;\mathrm{in}\;\mathrm{pellet}}{{\mathrm{Initial}\;\mathrm{amount}\;\mathrm{of}\;\mathrm{phthaloNH}}_{2}}\times 100$$

Knowing the size of one molecule of BSA (14 × 4 × 4 nm) and the volume of one BSA NP, we were able to determine the amount of BSA molecules per NP. Specifically, we calculated that in one NP we have 4074 BSA molecules. Then, we determined how many BSA NPs are in 1 mL of the final synthesis solution, using the following Equation (3):

(3) $$C(M)=\frac{\mathrm{md}}{M\;\cdot \mathrm{Vs}}$$

where C(M)—molar concentration; md—BSA mass (g); Vs—the solutions volume (L); and M—molar mass (g/mol).

Therefore, we obtain a number of 845 × 10^14^ BSA NPs in 1 mL of synthesis solution. Subsequently, using the same formula we determined a number of 164.400 × 10^14^ phthaloNH_2_ molecules in 1 mL of solution.

Finally, using the following Equation (4), we achieved a number of 195 phthaloNH_2_ molecules per one NP.

(4) $$\frac{{\mathrm{Number}\;\mathrm{of}\;\mathrm{phthaloNH}}_{2}\;\mathrm{molecules}}{1\;\mathrm{NP}}=\frac{{\mathrm{number}\;\mathrm{of}\;\mathrm{phthaloNH}}_{2}\;\mathrm{molecules}\;\mathrm{in}\;1\;\mathrm{mL}}{\mathrm{number}\;\mathrm{of}\;\mathrm{NPs}\;\mathrm{in}\;1\;\mathrm{mL}}$$

#### 2.2.3. Molecular Docking Simulation

Molecular docking is a useful tool in predicting the interactions that can take place between a ligand and a biomacromolecule. The molecular docking study was performed to obtain detailed information about how the phthaloNH_2_ molecule binds to albumin, theoretically investigating the potential interactions between them.

Herein, the reason behind choosing the two molecular docking software applications was to obtain and compare the results generated by different algorithms and scoring functions; thus, the result of the in silico experiment can be better cross-validated. To obtain a more accurate and realistic result from the molecular docking study, on both software applications used we have changed some computational parameters such as ga_run, exhaustiveness, and num_modes to higher values compared to their default ones (and with our previous report [27]). The results of the molecular docking studies performed using AutoDock and AutoDock vina were evaluated in terms of the binding affinity of phthaloNH_2_ to both Sudlow’s binding sites, I and II, expressed as the variation of the Gibbs free energy (ΔG) and the resulting inhibition constant (Table 1). For the resulted conformations, the mean ΔG and their standard deviation of the binding energies of the conformations were calculated and presented in Table 1. In addition, for both binding sites, using the built-in clustering function, the resulted 2 Å clusters of conformations of phthaloNH_2_ given by AutoDock were presented in Table 1.

The results of the molecular docking study performed with AutoDock indicate a good affinity of the compound phthaloNH_2_ for binding site I compared to site II. This is quantified by the value of ΔG = −10.79 kcal/mol for binding site I, better than that for site II, where ΔG = −8.80 kcal/mol. Consequently, it brings a much lower Ki value for site I (Ki = 12.23 nM) than for site II (Ki = 352.01 nM). Analyzing the dispersion of the binding energies of the generated conformations, we can observe that its average value in the case of site 1, ΔG = −10.78 kcal/mol, is very close to the best (ΔG = −10.79 kcal/mol), with a small energy dispersion (SD = 0.01) between all generated conformations. The conformations predicted for phthaloNH_2_ in site II have a slightly higher energy dispersion, the average value of ΔG = −8.80 kcal/mol, with a standard deviation equal to 0.05, slightly higher than in the case of site I. From a steric point of view, all conformations predicted of the phthaloNH_2_ molecule in site I are found in a 2 Å cluster, much better than in the case of site II, where the conformation with the best binding has in its cluster 294 conformations out of the 500 generated. The other 206 conformations are found in another two 2 Å clusters.

**Figure 4:**
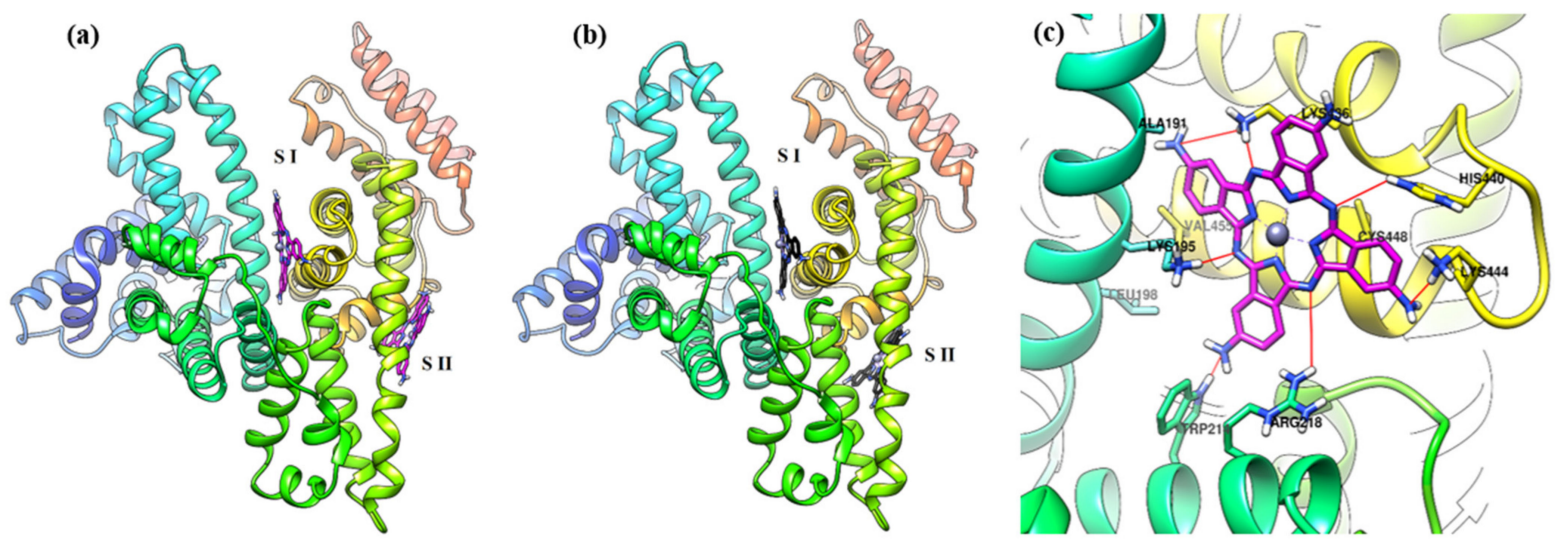
Graphic representation of the binding poses predicted for phthaloNH2 into the two Sudlow’s sites (S I and S II). The top conformations predicted by AutoDock are presented in the left side (**a**), with magenta colorization of the carbon atoms. The top conformations predicted by AutoDock vina are presented in the right side (**b**), with black colorization of the carbon atoms. The domains of albumin are colored the same between the two representations (**a**,**b**). The pose with the highest affinity of phthaloNH_2_ into Sudlow’s site I, with the depiction of interactions with the amino acid residues (**c**).

The results of the molecular docking study performed with AutoDock vina show the same trend as in the case of the previous results presented, obtained using AutoDock. PhthaloNH_2_ has a greater affinity for site I, rather than site II. This can be concluded by comparing its affinity for the two sites: ΔG = −11.10 kcal/mol for site I, and ΔG = −9.00 kcal/mol for site II, respectively. The energetic dispersion of the generated conformations indicates a high homogeneity of binding of phthaloNH_2_ to site I, analyzing this by means of the average binding energy and the standard deviation. The standard deviations of the conformation’s affinity are equal to 0.44 in the case of site I, while in the case of site II, it is slightly higher, equal to 0.49. The comparative representation of the best conformations predicted by the two software applications used for the molecular docking is presented in Figure 4. It can be seen that phthaloNH_2_ is positioned by both software applications in Sudlow’s site I in almost the same position, while in the Sudlow’s site II, they are significantly different.

Figure 4c depicts the predicted interactions that can take place between phthaloNH_2_ and the amino acids from albumin near Sudlow’s binding site I. All four nitrogen atoms from phthaloNH_2_ which connect the four isoindole units are predicted to interact with positively charged amino acid residues which surround the molecule (Lys195, Arg218, Lys436, and His440). Aromatic amines of phthaloNH_2_ are predicted to interact with Trp214, Lys444, and Lys436. Of these amino acids, Arg218, Lys436, and Lys195 are reported in the literature to be important for the interaction of albumin with ligands [34,35]. As phthaloNH_2_ is voluminous, it does not actually enter site I but is located in its vicinity, in the cleft found between subdomains IA and IIIA, as reported in the literature for the case of ligands with high molecular volume. PhthaloNH_2_ has a rigid structure and is extended in two dimensions, compared to other compounds that have a longitudinally extended appearance and possibly have elements of flexibility, allowing them to bend. Analyzing other reports in the literature, in which albumin has been co-crystallized with longitudinal-looking ligands or having flexible elements, deep binding of phthaloNH_2_ to Sudlow’s site I is difficult due to its size and rigidity [36,37].

Considering Sudlow’s site I as the main binding site of albumin, responsible for binding many drugs, and the preliminary docking results presented above, we will present a series of more detailed data from the molecular docking results of the compound at this site. Figure S4 shows the conformations with the highest affinity for site I, generated by the two software applications. It can be seen that they are almost overlapping. PhthaloNH_2_ adopts a propeller-like conformation, possible due to its planes of symmetry.

Both AutoDock and AutoDock vina have yielded consistent results following exactly the same trends; thus, we can conclude that phthaloNH_2_ binds well in site I of serum albumin, the conformations generated indicating that it binds homogeneously, taking into account the results from the energetic and steric perspective, as reported in the literature, as the main binding site of serum albumin for ligands. The contribution of site II in the binding of phthaloNH_2_ can be taken into account, but with negligible influence, due to the much lower affinity of phthaloNH_2_ to it and the dispersed conformations predicted into this site [38,39].

### 2.3. Free vs. Encapsulated PhthaloNH_2_ withdin BSA NPs

#### 2.3.1. Temperature-Dependent Spectroscopic Characterization

**Figure 5:**
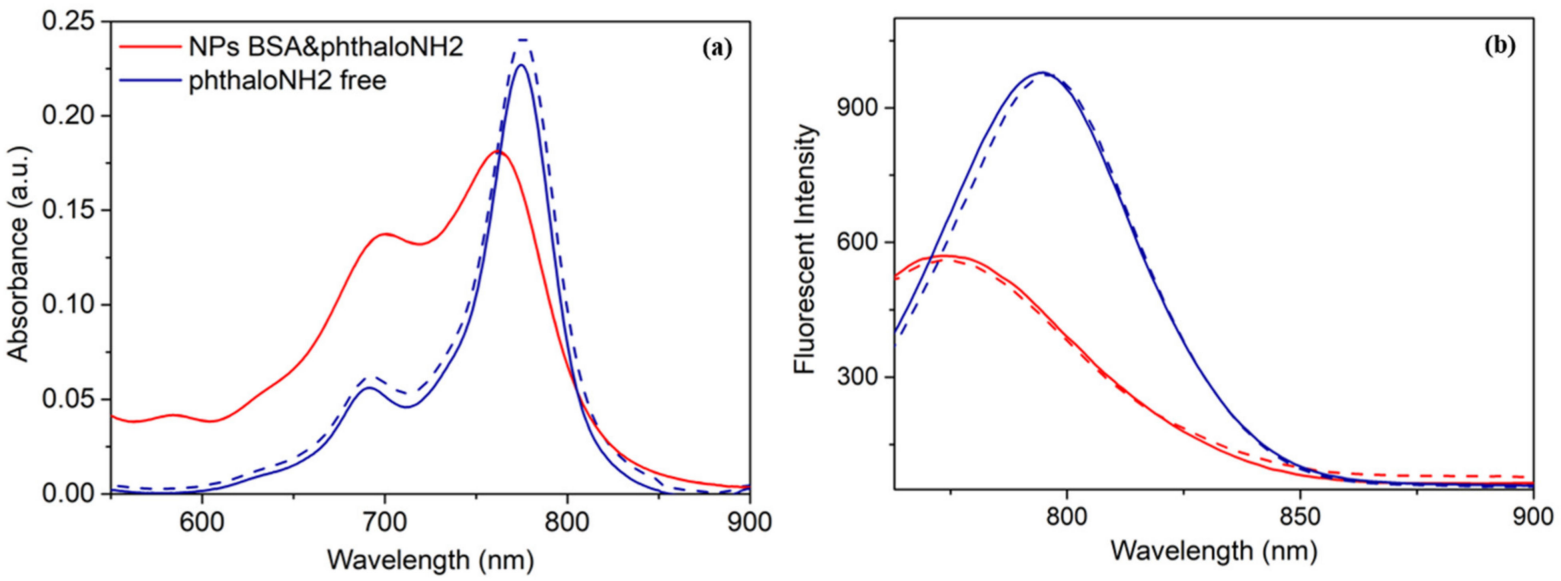
Absorption (**a**) and fluorescence emission (**b**) spectra recorded at 25 °C (dashed line) and at 37 °C (solid line) of the free photoactive agent (blue) compared with BSA&phthaloNH_2_ NPs (red).

Next, we wanted to compare the free phthaloNH_2_ (blue spectra) with the previously synthesized BSA&phthaloNH_2_ NPs (red spectra) at room temperature, 25 °C, and at a biologically relevant temperature, 37 °C, to confirm their adaptability in biomedical applications. In the absorbance (Figure 5a) and fluorescence emission (Figure 5b) spectra, we presented spectra measurements both at 25 °C and 37 °C. We can observe that the absorption and the emission maximum of BSA&phthaloNH_2_ NPs does not shift once the temperature is increased, remaining preserved both at 25 °C (dashed line) and 37 °C (solid line). On the one hand, in the absorbance spectra (Figure 5a), free phthaloNH_2_ has the exact same absorption maximum at 25 °C (dashed line) as at 37 °C (solid line), precisely 775 nm, but we can see a slight decrease in intensity as the temperature rises. On the other hand, in the fluorescence emission spectra, free phthaloNH_2_ does not maintain its emission maximum constant. At 25 °C, it has an emission maximum at 796 nm (dashed line), but at 37 °C, the emission maximum shifts to smaller wavelengths at 795 nm (solid line). Additionally, we can remark that the free phthaloNH_2_ does not lose its fluorescence emission intensity when the temperature rises. Moreover, the encapsulation of the phthaloNH_2_ agent in the protein-based NPs is confirmed with the BSA&phthaloNH_2_ NPs absorbance spectra change in the phthaloNH_2_ specific region, leading to a hypsochromic shift of 13 nm. BSA&phthaloNH_2_ NPs fluorescence emission undergoes changes compared to free phthaloNH_2_ because of the environmental change from the photoactive agent immediately surrounding once encapsulated within the BSA NPs. There is a 22 nm blueshift between the free photoactive agent and the BSA&phthaloNH_2_ NPs, proving once again that we successfully encapsulated the photoactive agent within the NPs.

#### 2.3.2. MTT Assay

**Figure 6:**
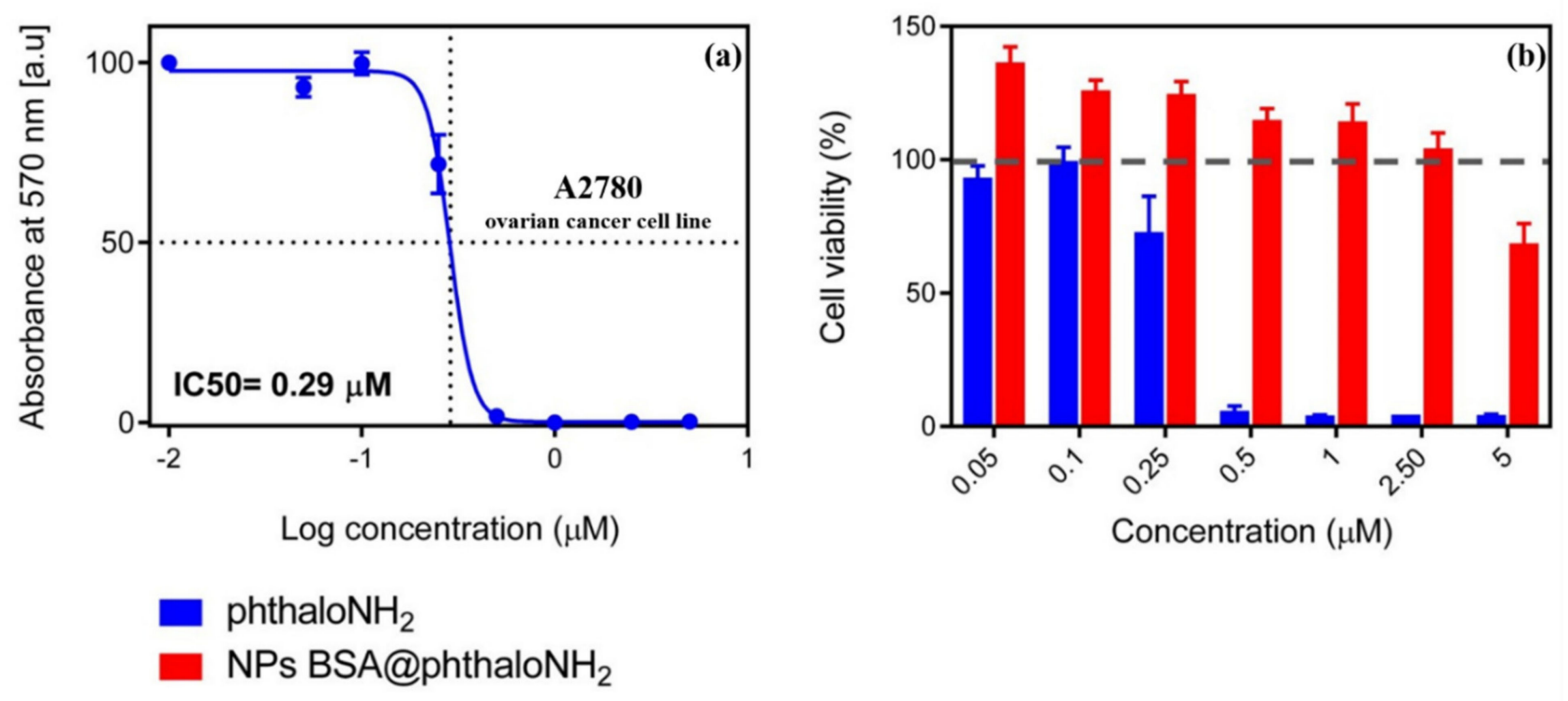
MTT cells assay; (**a**) survival curve of A2780 cells treated with phthaloNH_2_ —nonlinear regression; and four-parameter sigmoidal curve fit, each point representing mean ± SEM in three separate measurements; dose-dependent decrease of the viability of A2780 cells treated with phtaloNH_2_; (**b**) comparison of the viability of ovarian cancer cells treated with phthaloNH_2_ vs. BSA&phthaloNH_2_ NPs.

The MTT assay, the gold standard of the cell viability assay, has been the first step towards the translation of BSA&phthaloNH_2_ NPs to pre-clinical studies. Thus, the A2780 ovarian cancer cell responses to free phthaloNH_2_ and BSA&phthaloNH_2_ NP treatments were analyzed after a 24 h incubation period. A concentration-dependent decrease in the number of viable cells was observed for the cells treated with the free phthaloNH_2_ solution, and the IC50 value was calculated to be 0.29 µM (Figure 6a). On the other hand, the treatment with BSA&phthaloNH_2_ NPs led to no noteworthy variation in the viability of A2780 cancer cells, verifying their feasible utilization in biomedical applications. Furthermore, no cytotoxic effects were observed for the encapsulated phthaloNH_2_ molecule, even at concentrations 10 times higher than the IC50 values of the free phthaloNH_2_ solution (Figure 6b), underlining once again the biocompatibility of the BSA NPs and the necessity to load the photosensitizer within them.

#### 2.3.3. Photodynamic Effects

When irradiated, the PS molecule reaches the excited singlet state S_1_ due to photon absorption and returns to the ground state (S_0_) through various possible radiative (e.g., fluorescence) and non-radiative relaxation processes such as intersystem crossing to an excited triplet state level (T_1_) (Equation (5)). On the triplet state level, photochemical reactions are induced, which imply electron or hydrogen transfer. The PS reacts with ^3^O_2_ through energy transfer as the PS returns to the ground level and ^1^O_2_ is produced (Equation (6)), a strong cytotoxic agent in the PDT of cancers [40]. Hence, PDT efficiency depends on the yield of ^1^O_2_ generation but also on the nano-system ability to target the endorsed biological layer (for example, cancer cells) [4].

(5) $$PS\;\left(S_{0}\right)\overset{h\vartheta }{\to }\;PS\;\left(S_{1}\right)\overset{intersystem\;crossing}{\to }\;PS\;\left(T_{1}\right)$$

(6) PS T1+O 32 →PS S0+O 12 

For this purpose, we studied the ABDA molecule in the presence of both BSA&phthaloNH_2_ NPs and free phthaloNH_2_ for the detection of ^1^O_2_; photo-induced degradation of ABDA occurs due to its interaction with the generated ^1^O_2_. The evaluation principle of the photodynamic efficiency is based on the monitoring of ABDA degeneration throughout the irradiation period, the effect being recorded by a gradual decrease in intensity in the UV-vis absorbance region specific for ABDA.

**Figure 7:**
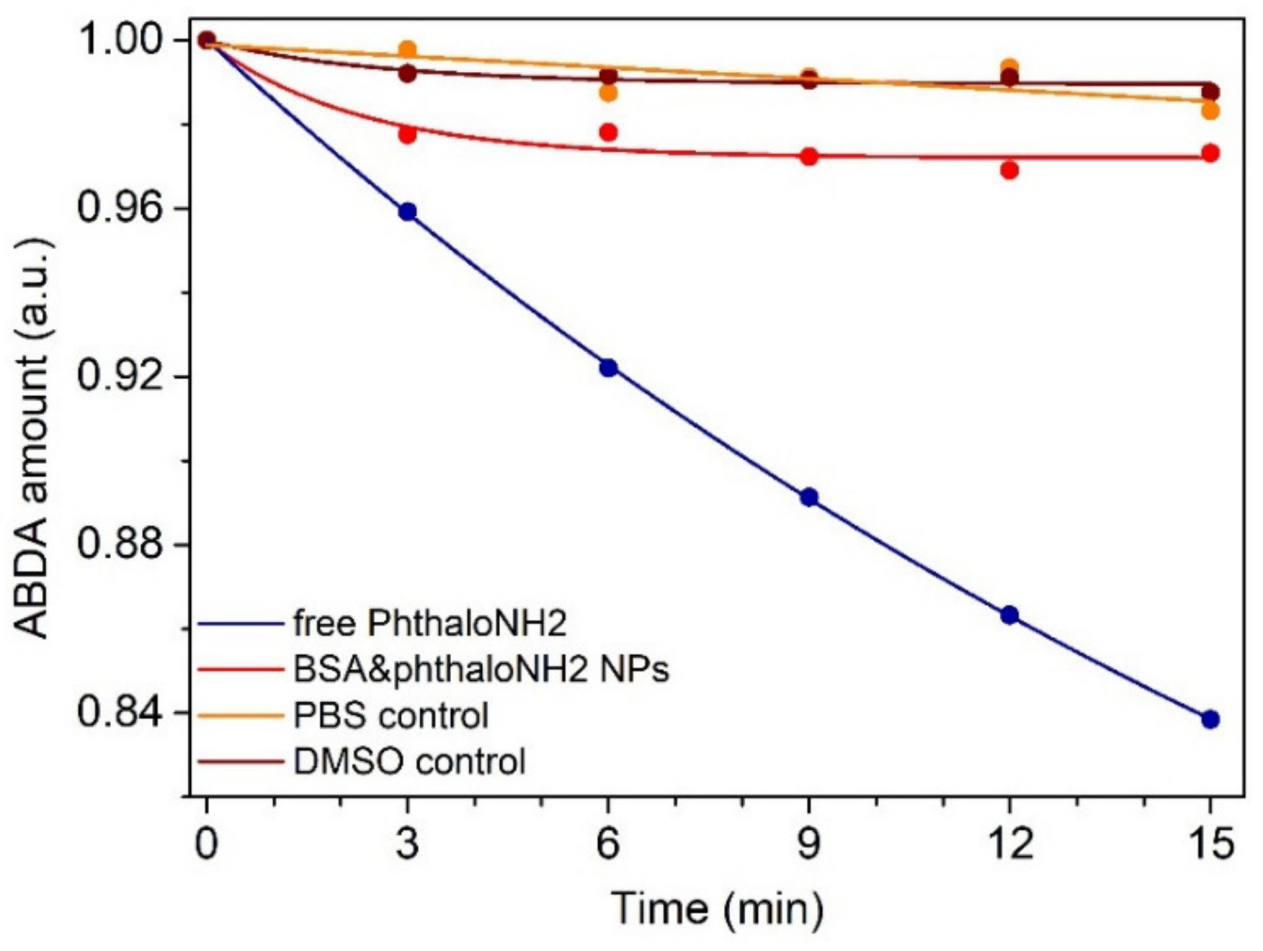
Degradation of ABDA molecule in the presence of free phthaloNH_2_ (blue), BSA&phthaloNH_2_ NPs (red), control PBS (orange), and DMSO (brown) solutions, monitored by modifications in the absorbance spectra of ABDA as a function of irradiation time.

To determine the efficiency to generate ^1^O_2_ of our BSA&phthaloNH_2_ NPs and free phthaloNH_2_, we analyzed the degradation of ABDA by monitoring its absorbance spectra (Figure S5) in the UV region every 3 min after irradiating with a 785 nm laser for a total time of 15 min. The BSA&phthaloNH_2_ NPs caused some degradation of the ABDA molecule (Figure 7 red spectra) in the first 3 min of irradiation, while little changes were observed in the absorbance spectra of ABDA for the rest of the irradiation period, resulting in an ^1^O_2_ quantum yield of 0.06. On the other hand, a constant decrease in the ABDA molecule amount was observed during the 15 min irradiation in the presence of the free phthaloNH_2_ (Figure 7, blue spectra), resulting in an ^1^O_2_ quantum yield of 0.22. The discrepancies in the ^1^O_2_ quantum yield values determined for the two samples can be associated with the phthaloNH_2_ molecule’s rigid structure and possible hydrogen bridge formation once it binds within the BSA protein. In addition, closely related to the changes in the configuration of the molecules is another key property of PS agents, namely their intrinsic intersystem crossing rate. When encapsulated within the BSA protein, the dye is sheltered from the environment, and compared to the free phthaloNH_2_ solution, the phthaloNH_2_–^3^O_2_ interaction probability is lower. Another important factor in the dual behavior of phthaloNH_2_ is the preferred solvent for each sample during the PDT assay, PBS for the BSA&phthaloNH_2_ NPs and DMSO for the free phthaloNH_2_, considering the hydrophobicity of the dye and the degradation of the BSA NPs in DMSO. Control PBS and DMSO solutions were studied under identical conditions, but no indications of ^1^O_2_ generation were observed.

#### 2.3.4. Photothermal Effects

To validate the protein-based NPs’ potential as therapeutic agents in solution, we tested their capacity to convert light to heat and compared it with the one of the free phthaloNH_2_ after irradiation with the same NIR laser, starting from 37 °C. The second non-radiative relaxation process relies on the internal conversion from the S_1_ excited state in the form of heat and is known as the photothermal effect. The photothermal effect is described by the energy balance equation (Equation (7)); by analytically solving this equation, we can determine the temperature dissipated by the system to the surrounding environment (Equation (8)) [4].

(7) $$\sum m_{i}C_{i}\frac{dT}{dt}=Q_{0}-Q_{ext}$$

(8) ΔT=τQ0∑miCi(1−e−tτ)

**Figure 8:**
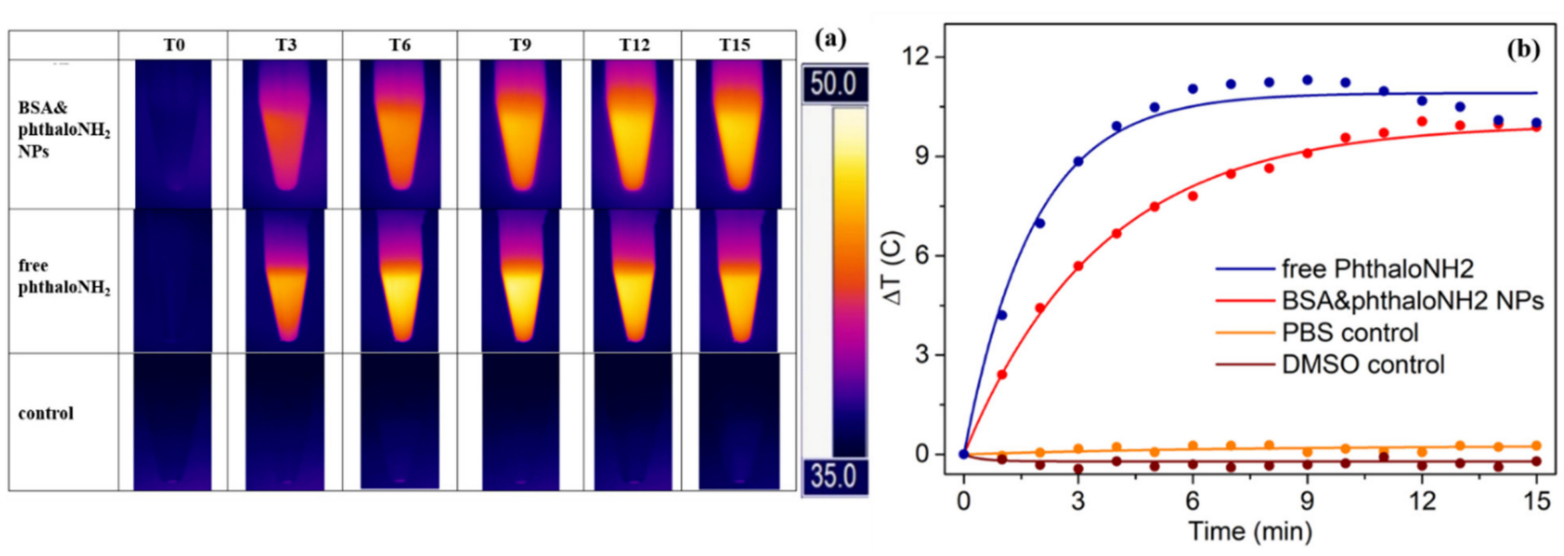
(**a**) Real-time photothermal images recorded under 785 nm laser irradiation through 15 min every 3 min of BSA&phthaloNH_2_ NPs, free phthaloNH_2_, and PBS (as control); (**b**) Heating curves of free phthaloNH_2_ (blue), BSA&phthaloNH_2_ NPs (red), control PBS (orange), and DMSO (brown) solutions plotted against 15 min irradiation time.

Thus, to assess the efficiency of our fluorescent protein-based NPs to transform light to heat, as thermal agents in cancer therapy, we performed photothermal experiments on BSA&phthaloNH_2_ NPs, free phthaloNH_2_, and control solutions such as PBS and DMSO for comparison. From Figure 8a,b, it can be observed that the BSA&phthaloNH_2_ NPs heat up gradually, starting from an ambient temperature of 37 °C and reaching temperatures around 47 °C after just 15 min. These results confirm the promising potential of the BSA&phthaloNH_2_ NPs to be translate to pre-clinical studies, considering that their maximum temperature achieved is in the optimal temperature-time range for moderate-temperature hyperthermia [41].

Next, the photothermal conversion efficiency (η) of the BSA&phthaloNH_2_ NPs was calculated to be 20%. The fact that these particles heat up with approximately 10 °C in such a short irradiation period and taking into account their great light-to-heat conversion performance, BSA&phthaloNH_2_ NPs show promising results in our search for obtaining NIR protein-based NPs with dual therapeutic effect. Compared to BSA&phthaloNH_2_ NPs, the free photoactive agent reaches a maximum temperature of 48.16 °C after the first 9 min of irradiation, but until minute 15, the temperature decreases to 46.87 °C. The free phthaloNH_2_ solution reaches slightly higher temperatures as the BSA&phthaloNH_2_ NPs because, once encapsulated, the photoactive molecule is protected within the protein, the heat transfer is lower, and it interacts less with the irradiation light. Nonetheless, the performance of the tested samples is determined based on the light-to-heat conversion efficiency. Thus, we can conclude that the BSA&phthaloNH_2_ NPs present better outcomes in comparison to the free phthaloNH_2_ solutions, the η for the free phthaloNH_2_ solution being 8.5%, once again underlining the benefit of encapsulating the NIR photoactive agent within BSA NPs. Contrary to the values of maximum temperature reached during the 15 min experiments, the light-to heat conversion efficiency is higher for the BSA&phthaloNH_2_ NPs due to the significant contribution of the absorption correction factor calculated for the optical density at the excitation wavelength (optical density at 785 nm for free phthaloNH_2_ solution: 0.08, and for BSA&phthaloNH_2_ NPs: 0.05) parameter present in the photothermal conversion efficiency equation (Equation (10), Section 4.8). Another key element in the discrepancy between the maximum temperature values and conversion efficiencies is the great difference in the specific heat capacity of the selected solvents; 1.97 J g^−1^ K^−1^ for DMSO (phthaloNH_2_@DMSO) and 4.18 J g^−1^ K^−1^ for PBS (BSA&phthaloNH_2_ NPs@PBS). It is worth noting that the PBS and DMSO solutions are not photothermally active.

#### 2.3.5. Photothermal and Size Stability

**Figure 9:**
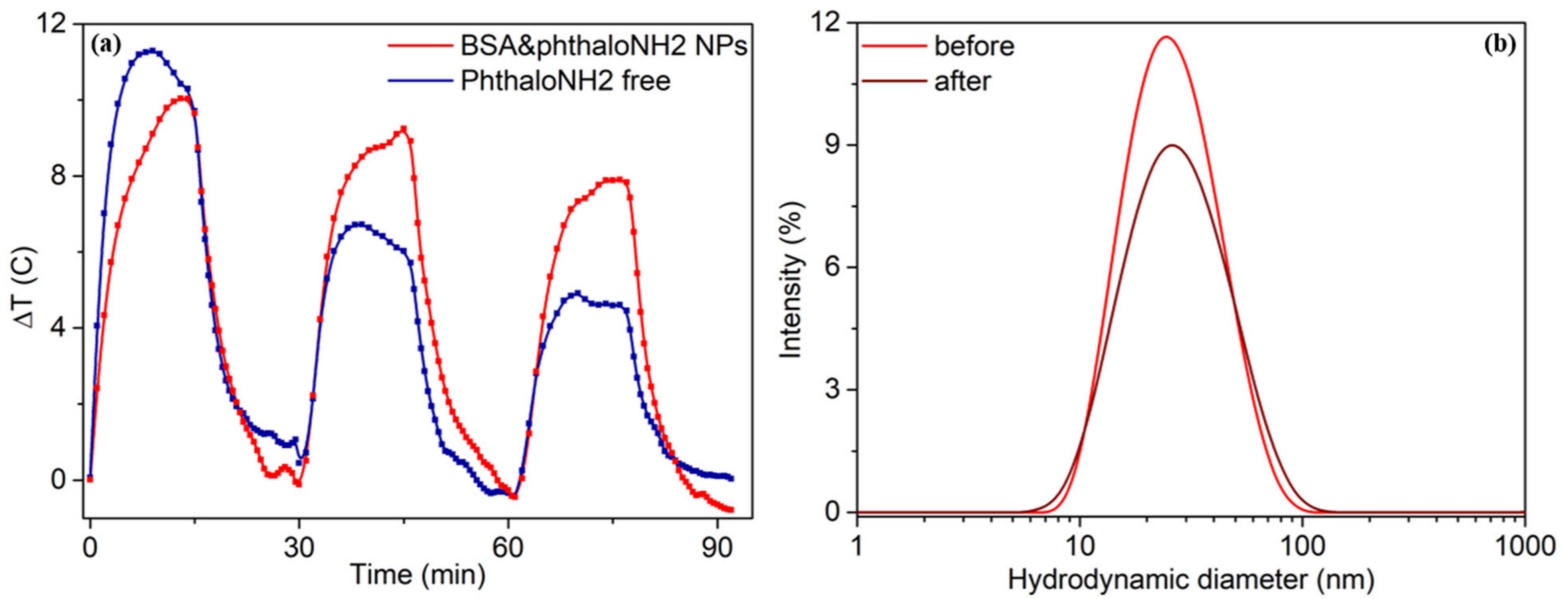
(**a**) The thermal curves of the BSA&phthaloNH_2_ NPs (red) and of the free phthaloNH_2_ (blue) obtained after three On/Off irradiation cycles. (**b**) The DLS spectra present BSA&phthaloNH_2_ NPs’ hydrodynamic diameter stability assay before (red) and after (brown) irradiation.

Later on, to study the photothermal stability of the BSA&phthaloNH_2_ NPs and of the free phthaloNH_2_, we exposed them to three successive heating and cooling down (On/Off) cycles. We exposed the samples to the laser for 15 min and then turned it off for another 15 min, allowing them to cool down to ambient temperature. As shown in Figure 9a, free phthaloNH_2_ (blue spectra) is able to convert light to heat faster, but after the first On/Off cycle, thermal losses are observed. In the second cycle, the free photoactive agent heats up to 59.7% of the maximum temperature reached, while the NPs heat up to approximately the same temperature of 47 °C, precisely 91.45% of the maximum temperature recorded in the first cycle. In the last cycle, as also shown in Figure 9a, the maximum temperature of the free phthaloNH_2_ was reduced by 55.8%, whereas for the BSA&phthaloNH_2_ NPs, the maximum temperature was better maintained, being decreased by only 32.1%. This is another proof that we successfully encapsulated and protected the photoactive agent within the NPs.

Subsequently, we studied the BSA&phthaloNH_2_ NPs’ hydrodynamic diameter before and after irradiation. From Figure 9b, we observe that the BSA&phthaloNH_2_ NPs decrease in intensity after irradiation, but the hydrodynamic diameter remains relatively constant. Before irradiation, the protein-based NPs have a hydrodynamic diameter of 28.54 ± 0.42 nm and PDI 0.194 ± 0.027, and after exposure to the NIR laser line, they present a hydrodynamic diameter of 30.66 ± 1.30 nm and PDI 0.185 ± 0.062, the insignificant 2 nm increase upholding the good size stability of our BSA&phthaloNH_2_ NPs.

## 3. Discussion

To conclude, in this article, we successfully synthesized new and highly reproducible NIR albumin-based NPs as dual phototherapeutic agents, with versatile surface and great post-irradiation size stability NPs, having a diameter confirmed both by DLS and TEM images of 25.06 ± 0.62 nm. The successful encapsulation of the free NIR photoactive agent within the NPs is confirmed both by molecular docking simulations, indicating the binding of the phthaloNH_2_ molecule in site I of the albumin molecule, and also by the BSA&phthaloNH_2_ NPs’ absorbance spectra change in the phthaloNH_2_ specific region. Furthermore, we demonstrated the NPs’ stability at different temperatures, with a preserved absorption and emission both at 25 °C and 37 °C. We validated the BSA&phthaloNH_2_ NPs’ slight capacity to generate singlet oxygen by monitoring the degradation of the ABDA sensor under irradiation with a NIR 785 nm laser, resulting in a ϕ(^1^O_2_) of 0.06. The photothermal assay performed in the presence of the BSA&phthaloNH_2_ NPs was a success, with an increase of 10 °C after only 15 min of irradiation with the 785 nm NIR laser, compared to the free phthaloNH_2_, which increases its temperature by 11 °C after 9 min of irradiation but then reaches a saturation point, and by the 15th minute of irradiation, its temperature is decreased by 2 °C. The BSA&phthaloNH_2_ NPs present great photostability after 3 On/Off irradiation cycles (reaching 67.9% of the maximum temperature recorded in the first cycle), compared to the free phthaloNH_2_ which heats up only to 44.2% of the maximum temperature attained in the first cycle. To summarize, in this article, we reported the potential of our freshly synthesized BSA&phthaloNH_2_ NPs to be translated in the future as therapeutic and fluorescent contrast agents in pre-clinical and clinical settings, by employing their great particle yield (over 75%), terrific encapsulation efficiency of phthaloNH_2_ (over 91%), biocompatibility proven by the MTT assay, and surface capacity to be further functionalized for target delivery, alongside their dual photodynamic-photothermal therapeutic effects that could be implemented in the ongoing fight against cancer.

## 4. Materials and Methods

### 4.1. Materials

The materials used for this study are the following: bovine serum albumin; glutaraldehyde solution—grade I, 25% in water; 9,10-anthracenediyl-bis(methylene)dimalonic acid (ABDA); sodium sulfide nonahydrate (Na_2_S·9H_2_O); dimethylformamide (DMF); hydrochloric acid (HCl); sodium hydroxide (NaOH); RPMI medium, fetal calf serum (FCS); l-glutamine, penicillin-streptomycin solution; and [3-(4,5-dimethylthiazol-2-yl)-2,5-diphenyl-2H-tetrazolium bromide] (MTT). These were purchased from Sigma-Aldrich (Saint Louis, MO, USA) and used without any additional purification. Ethyl alcohol (EtOH) and dimethyl sulfoxide (DMSO) were purchased from Nordic Chemicals (Cluj-Napoca, Romania) and phosphate-buffered saline (PBS), without calcium or magnesium, was purchased from Lonza (Basel, Switzerland). We obtained ultrapure water from the Milli-Q purification system (Merck Millipore, Burlington, MA, USA), and we used it for all aqueous solution. The photoactive molecule of interest—phthaloNH_2_—was synthesized at the University of Chemistry, Cluj-Napoca, Romania (details in Section 4.2). For cell viability studies, A2780 ovarian cancer cell line from The European Collection of Authenticated Cell Cultures (ECACC), Porton Down Salisbury (Wiltshire, UK), was evaluated.

### 4.2. Synthesis of PhthaloNH_2_

The Zinc(II) tetranitrophthalocyanine (0.5 g × 0.6 mmol) and Na_2_S 9H_2_O (2 g, 8.3 mmol) were added to a 50 mL flask containing 10 mL DMF, stirred, and heated at 60–65 °C for 10 h, while the reaction progress was monitored by thin layer chromatography (TLC). The reaction mixture was cooled at room temperature, and after that, 10 g of crushed ice were added and stirred until melted. The solid was removed by filtration and was purified 3 times by mixing with 50 mL HCl 1M at 95 °C for 30 min. Then, it was filtered and washed with water until neutral pH. The solid was mixed with 50 mL NaOH at 90 °C for 30 min, filtered, washed with water until pH was neutral, and, finally, washed with ethanol, yielding 0.19 g (η = 50%) of dark greenish solid. MS (MALDI-TOF/TOF) *m*/*z*: 638.119 calculated for C_32_H_20_N_12_Zn, found 638.598 (Figure S6). FT-IR (KBr) v_max_/cm^−1^: 3405, 1612, 1522, 1416, 1339, 1149, 1119, 1100, 1089, 1046, 848, 780, 757.

### 4.3. Design of BSA&phthaloNH_2_ NPs

**Scheme 1:**
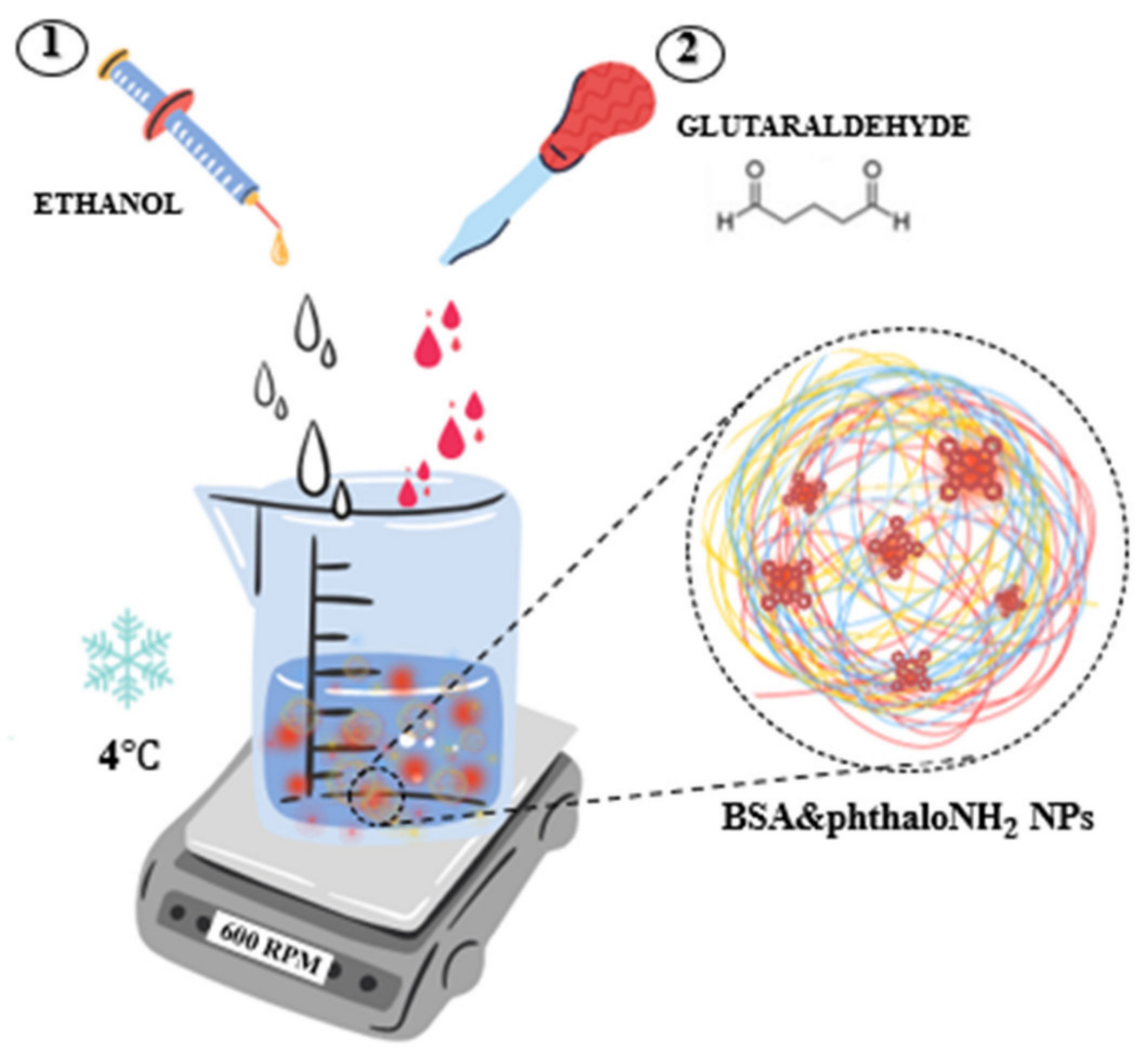
The synthesis protocol of BSA&phthaloNH_2_ NPs consisting of two steps: (i) desolvation and (ii) cross-linking.

One major challenge presented in the literature in developing therapeutic agents is the synthesis of NPs with desired properties and functions. In many cases, the physical and chemical properties, such as the applicability of therapeutic NPs, are critically controlled by their size and surface chemistry, as well as the embedded photoactive molecule quantity. Thus, the first step of this study is focused on developing, in a controlled and reproducible manner, fluorescent protein-based NPs through loading the newly synthesized photoactive molecules with fluorescence emission in the NIR region, namely phthaloNH_2_. These NPs, being further referred to as BSA&phthaloNH_2_ NPs, were synthesized on an ice bed after a modified and optimized synthesis protocol previously reported by our group [27] consisting of two steps: (i) desolvation using an organic solvent, ethanol, and (ii) stabilization by chemical cross-linking using glutaraldehyde (Scheme 1).

We began by dissolving the BSA protein in water (5% concentration), having an adjusted pH of 8. Then, we obtained a solution based on EtOH and 100 μL phthaloNH_2_ stock solution, which was added in drops (1:1) over the BSA in water with a controlled speed of 1 mL min^−1^ using an automated syringe pump system, specifically NE-4000 Programable Syringe from NEW ERA Pump systems Inc. (New York, NY, USA). The resulted solution was stirred during the entire preparation on a magnetic stirrer set at 600 RPM. After another 15 min, step 2 follows: the addition of glutaraldehyde as the stabilizer agent over the initial mixture, and finally, the solution was stirred for 24 h at a 4 °C temperature. The quantity of glutaraldehyde, in our case 40%, was relatively calculated as a function of the theoretical quantity needed for cross-linking all of the amino groups. The obtained NPs were firstly purified through a mechanic filter of 220 nm and then centrifuged for 1 h at room temperature, at 6000 RPM, in concentration tubes with a 100 kDa filtration membrane, to eliminate all remaining protein molecules after the NPs were formed. Subsequently, the solution was suspended in its initial volume in PBS. To eliminate the free dye excess, the NPs were centrifuged for 3 h at 18,000 RPM at room temperature in 1.5 mL centrifuge tubes. The supernatant was collected and stored for further use, while the pellet was suspended in DMSO and measured to determine the phthaloNH_2_’s loading efficiency in NPs.

### 4.4. Particle Yield and Dye Loading Efficiency

To establish the particle yield and dye loading efficiency after the NPs were formed, we analyzed the final BSA and photoactive molecule concentration through monitoring the absorbance of the BSA&phthaloNH_2_ NPs in the UV region for BSA (190–350 nm) and also in the NIR region for the phthaloNH_2_ from the pellet (550–900 nm). In parallel, we measured the absorbance of different concentrations of BSA solutions, 1, 2, 3, 4, and 5%, and phthaloNH_2_ resuspended in DMSO solutions, 7.5 × 10^−7^, 10^−6^, 2.5 × 10^−6^, 7.5 × 10^−6^, 10^−5^, and 2.5 × 10^−5^ M, in order to create calibration curves.

### 4.5. Molecular Docking

Serum albumin, a kind of globular protein from the family of albumins, can bind and transport a wide variety of substances in human plasma. It has multiple binding sites with different affinities for ligands, due to various hydrophobicity and spatial structure of the ligands and binding pockets [42]. The most common approach used in determining the ligand binding manner to proteins is the molecular docking study [43,44]. Next, we investigated the molecular mechanism of interaction between phthaloNH_2_ and serum albumin, taken from RCSB Protein Data Bank (entry code: 1AO6) [45,46]. The files of phthaloNH_2_ and macromolecules required for performing the molecular docking were prepared using the standard previously reported procedure using AutoDock Tools 1.5.6 and OpenBabel 2.3.2 [47,48,49,50].

The molecular docking of phthaloNH_2_ to serum albumin was carried out based on our group previous report using AutoDock 4.2.6 [49]. To have a clearer image about the molecular interactions that can take place between the phthaloNH_2_ and serum albumin, a supplementary molecular docking software, AutoDock vina 1.1.2, was used [51].

We have targeted in the molecular docking study the sites responsible for the carrier role of the protein, known as Sudlow’s sites I and II, to determine the preferred site in which the compound binds. Both sites were found in the A fragment of the deposited homodimer structure. The present molecular docking study did not evaluate the binding of phthaloNH_2_ to another possible site: for example, at the surface of albumin macromolecules. As the albumin macromolecules were aggregated together in the process of synthesis of the nanoparticles, they have mutual influences, such deformations of steric hindrance on the external surfaces and pockets, making the molecular docking study unrealistic from this point of view. However, inclusion between two or more albumin macromolecules of a phtaloNH_2_ molecule may be possible but difficult to evaluate by molecular docking studies. The search space was configured for the docking procedure as specifically targeting the two pockets and avoiding blind docking [43]. The search space was configured as a cube, with coordinates of the center, as previously reported by our team [27]. In order to acquire more precise results, we changed some of the default or previously reported parameters in the molecular docking study. For AutoDock, the parameter ga_run was set to 500, and for AutoDock vina, parameters exhaustiveness and num_modes were set to 30 and 25, respectively. The conformations generated by AutoDock in both binding sites were grouped in clusters with relative root mean square deviation of 2 Å using the built-in function. The visualization of the docking results was performed using Chimera 1.10.2 [52].

### 4.6. Cell Viability

At this point we focused on the viability of A2780 ovarian cancer cell line, in the presence of free phthaloNH2 and BSA&phthaloNH2 NPs. More specifically, the cells were kept in a humid incubator with 5% CO_2_ at 37 °C in RPMI medium with 10% FCS, 1% glutamine, and 1% penicillin-streptomycin. A colorimetric cell viability assay, namely MTT, was used in order to assay the toxicity of free phthaloNH2 and BSA&phthaloNH2 NPs against A2780 cells. On 96-well plates, 20,000 cells/well were seeded and serial dilutions (0.05, 0.10, 0.25, 0.50, 1, 2.50, 5 μM) of both samples were added, while three wells were left without treatment, as control. Twenty-four hours later, the cellular media was changed, and MTT solution was added (100 μL, 1%). The cells were incubated for another hour until formazan crystals were formed, and 150 μL of DMSO was added to dissolve them. A Synergy 2 microplate reader from BioTek (Winooski, VT, USA) was used to measure the absorbance at 570 nm, and the surviving fraction was determined as the absorbance of the sample/absorbance of non-treated cells × 100. All experiments were executed in triplicate, and GraphPad Prism 6 software (San Diego, CA, USA) was implemented to process all data.

### 4.7. Photodynamic and Photothermal Effects

**Scheme 2:**
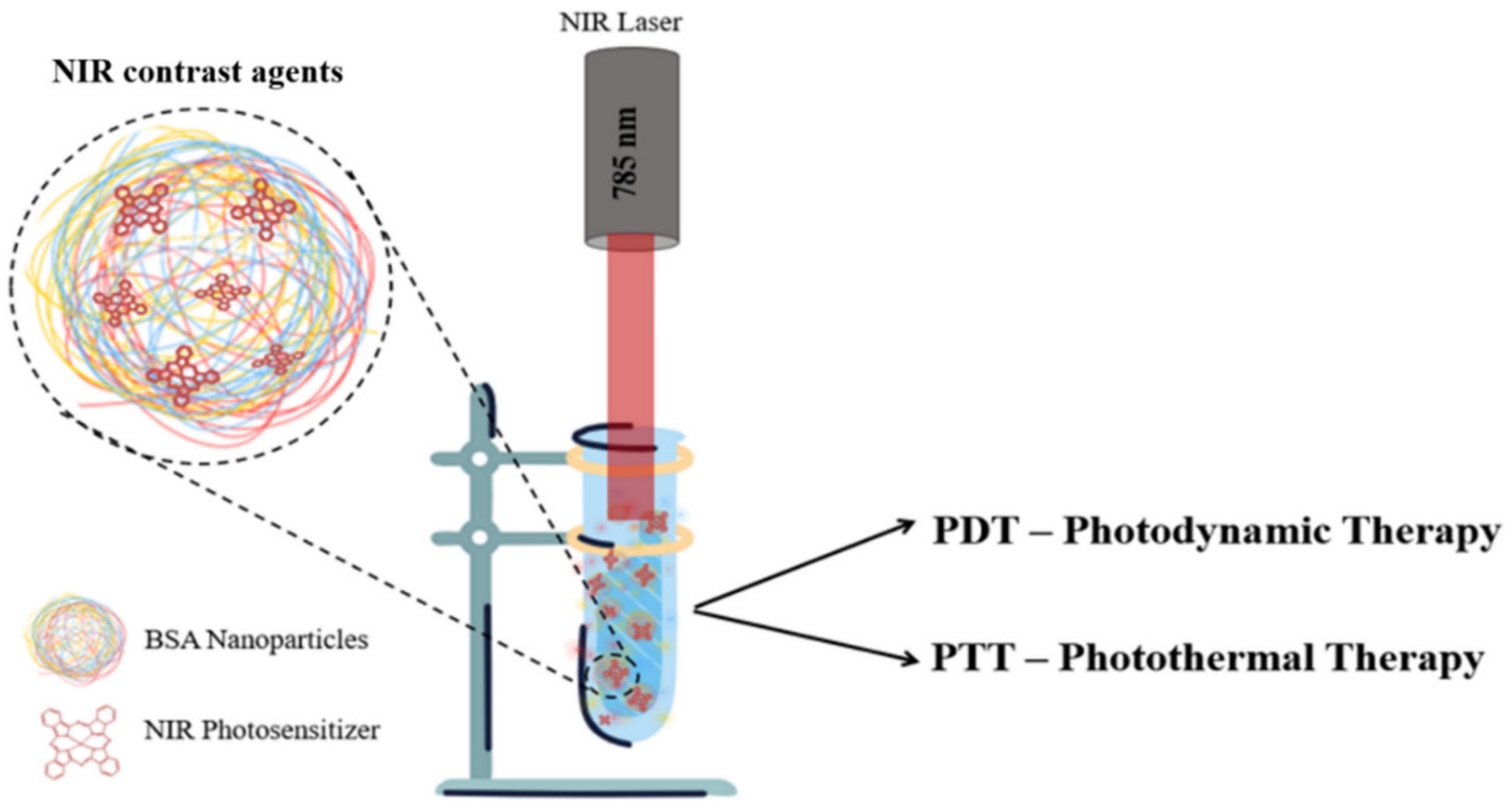
Illustration of the freshly synthesized NPs’ potential operating as dual therapeutic agents upon irradiation with NIR laser.

The next step was the evaluation of the therapeutic potential of the BSA NPs loaded with photoactive molecules through testing the photoactive agents’ capacity to generate singlet oxygen species (^1^O_2_) in solution after irradiation with a NIR laser (Scheme 2), starting from the biologically relevant temperature of 37 °C.

Specifically, we started by measuring the UV-vis absorbance spectra (T0) of 500 μL of sample in the presence of ABDA (0.3 mM) at 37 °C in a chamber. Then, we irradiated the sample with continuous-wave laser (i.e., laser diode with 785 nm wavelength of a Raman portable spectrometer R-3000CN, Raman Systems), at a power of 190 mW (0.93 W/cm^2^ intensity) for 3 min and measured the absorbance spectrum again; these procedures were repeated 5 times. All UV-vis absorbance spectra were studied, and the intensity in the maximum point of the ABDA specific absorbance band located at 380 nm was recorded. In addition, control samples were tested under identical experimental conditions.

The PDT performance of the free phthaloNH_2_ and BSA&phthaloNH_2_ NPs was determined by calculating the singlet oxygen quantum yield using Equation (9) [4]:

(9) ΦO 12=ΦO 12MB* S*FMBSMB*F

where ϕ(^1^O_2_)—the oxygen singlet quantum yield of the investigated sample; ϕ(^1^O_2_)^MB^—the known quantum yield of the reference molecule (in our case, we used methylene blue (MB) as reference, whose ϕ(^1^O_2_)^MB^ = 0.52 [4,52]); S—slope of the absorbance difference of ABDA in function of time for both sample and reference probe; and F—the absorption correction factor determined as F = 1 − 10^−OD^, where OD is the optical density at the excitation wavelength, in our case at 785 nm.

For the PTT evaluation, we maintained the same experimental setup and parameters as for PDT; 350 μL of each sample was exposed continuously for 15 min to the 785 nm laser line, while every 30 s, we collected thermal images obtained with an IR camera with thermal vision, namely Optris PI 450 (Remscheid, Germany). After 15 min of irradiation, we turned off the laser and allowed the samples to cool down to ambient temperature. From the recorded thermal images, we extracted the temperature at every minute during the irradiation, thus determining the temperature increase relative to the ambient temperature. The photostability of the free phthaloNH_2_ and BSA&phthaloNH_2_ NPs was monitored during 3 On/Off cycles, while DLS measurements were recorded both before and after the irradiation to test their morphological stability.

To study the photothermic effect of the BSA&phthaloNH_2_ NPs, we made DLS measurements both before and after irradiating them.

We determined the photothermal conversion efficiency (η) for both the free phthaloNH_2_ and BSA&phthaloNH_2_ NPs using Equation (10) [4]:

(10) $$\eta =\frac{h*A\Delta T-I\zeta }{I\left(1-\zeta \right)\left(1-10^{-A_{\lambda }}\right)}$$

where h—the heat transfer coefficient; A—the area cross section of irradiation; ΔT—the temperature difference; I—the incident laser power; ζ—the energy fraction absorbed by the cuvette and solvent; and A_λ_—the absorbance of the probe at the excitation wavelength (more specifically, 785 nm).

### 4.8. Characterization Techniques

Mass spectrometry was performed on a Rapiflex MALDI-TOF from Bruker Daltonics (Bremen, Germany) equipped with a Smartbeam 3D laser. FlexControl Version 4.0 from Bruker Daltonics was used to optimize and acquire data using the following parameters: positive ion polarity in reflectron mode, mass scan range (*m*/*z* 100–1600), digitizer (5 GHz), detector voltage (2087 V), 1000 shots per pixel, and 10 kHz laser frequency. The laser power was set at 50% to 80% of the maximum, and 5000 laser shots were accumulated for each spectrum. The compound was dissolved in DMSO, and then a sample solution (1 μL) was deposited onto the MALDI target, dried at room temperature, and analyzed in MALDI-TOF-MS using DHB (2,5-dihydroxybenzonic acid) without using MALDI matrix. Mass calibration of MALDI-TOF-MS was performed by the peptide mixture standard solution from Bruker Daltonics. TLC was performed on silica gel 60 F_254_ aluminum sheets from Merck Millipore.

The FTIR spectra were recorded in KBr pastille using Alpha II spectrometer from Bruker Daltonics.

The UV-vis-NIR absorption spectra measurements were carried out with a Jasco V-670 UV-vis-NIR spectrophotometer procured from Jasco International Co., Ltd. (Tokyo, Japan) at a spectral resolution of 1 nm. The measurements were performed at room temperature as well as at 37 °C (relevant temperature from biological point of view) with the Peltier thermostatted single cell holder (air cooled), using quartz glass cuvettes (optical path of 2 mm) from Hellma (Müllheim, Germany).

The fluorescence emission measurements were performed by Jasco FP6500 spectrofluorometer from Jasco International Co., Ltd. (Tokyo, Japan). All measurements were performed at room temperature as well as at 37 °C with the Peltier thermostatted depolarization accessory (water-cooled), with a spectral resolution of 1 nm and a Xenon lamp as the excited source of 150 W. Emission spectra were obtained at a fixed excitation wavelength of 745 nm and 1 × 10 nm excitation and emission bandwidths, using quartz glass cuvettes of 5 × 5 mm from Hellma (Germany). The results have been recorded and analyzed using Spectra Manager program. We used the same concentration (herein 10^−5^ M) of phthaloNH_2_ for both free phthaloNH_2_ and the loaded synthesized NP solutions.

Dynamic light scattering (DLS) and zeta potential measurements helped us to determine the hydrodynamic diameter and surface potential of the BSA&phthaloNH_2_ NPs. All measurements were made in triplicate using Zetasizer NanoZS90 equipment from Malvern Panalytical Ltd. (Worcestershire, UK) and using micro cuvettes for single use. In addition, for zeta potential measurements, we used a *dip cell* set (ZEN1002), and we applied a 5 V voltage. The equipment has a He-Ne laser (633 nm, 5 mW), and all measurements were recorded at fixed temperatures of 25 °C and 37 °C.

Transmission electron microscopy (TEM) imaging was employed to determine the dry size of the BSA&phthaloNH_2_ using a JEOL JEM-1010 microscope (Tokyo, Japan). The accelerating voltage was set at 30 kV, and the NPs were diluted in water (1:5000), dispersed on cellulose coated grids, and negatively stained by using uranyl acetate.

## Figures and Tables

**Table 1:** The results of the docking study expressed as binding affinity of phthaloNH_2_ to site I and site II of serum albumin expressed as variation of Gibbs free energy (ΔG), the computed inhibition constant (Ki), and the analysis of clusters.

Sudlow’s Sites	AutoDock							Autodock Vina		
	ΔG (kcal/mol)	Ki (nM)	Mean ΔG (kcal/mol)	ΔG SD	NC	Other Clusters		ΔG (kcal/mol)	Mean ΔG (kcal/mol)	ΔG SD
						NCoC	NoCl			
Site I	−10.79	12.23	−10.78	0.01	500	0	0	−10.10	−9.50	0.44
Site II	−8.80	352.01	−8.73	0.05	294	206	2	−9.00	−7.95	0.49

ΔGSD—standard deviation of the binding affinity of the resulted conformations; NC—number of conformations; NCoC—number of conformations found in other clusters; NoCl—number of other clusters.

## Data Availability

The data presented in this study are available on request from the corresponding author.

## Supplementary Materials

The following are available online, Figure S1: Zeta potential measurements of the BSA&phthaloNH2 NPs at 25 °C (solid line) and at 37 °C (dashed line), Figure S2: (a) DLS, (b) fluorescence, and (c) absorption spectra of the BSA&phthaloNH2 NPs just after development (red) and after 3 months of storage (pink), Figure S3: The calibration curves for different concentrations of (a) free BSA (b) phthaloNH2 dye solution, Figure S4: View from both sides of the poses predicted for phthaloNH2 into the two Sudlow’s site I by (a) AutoDock (carbon atoms in magenta) and (b) AutoDock vina (carbon atoms in black). Some amino acid labels were depicted for better comprehension, Figure S5: Degradation of ABDA during 15 min of irradiation in the presence of (a) BSA&phthaloNH2 NPs and (b) free phthaloNH2, Figure S6: Mass spectra (MALDI-TOF/TOF): (a) the DHB (2,5-dihydroxybenzoic acid) matrix and (b) Zn (II)-2,9,16,23-tetraaminophthalocyanine (m/z = 638.578) in the presence of the DHB matrix.

## Sample Availability

Samples of the compounds are available from the authors.
