# Supplementary material for: Fluorescent Phthalocyanine-Encapsulated Bovine Serum Albumin Nanoparticles: Their Deployment as Therapeutic Agents in the NIR Region
Source: Molecules. 2021 Aug 2;26(15):4679. doi: 10.3390/molecules26154679 (PMC8348139; doi:10.3390/molecules26154679)
Supplement: Supplementary file 1 [file molecules-26-04679-s001.zip › molecules-1301540-supplementary.pdf]

Fluorescent phthalocyanine-encapsulated bovine serum albumin nanoparticles: their deployment as therapeutic agents in the NIR region

Raluca Borlan[1,2], Daria Stoia[2], Luiza Gaina[3], Andreea Campu[2], Gabriel Marc[4], Maria Perde-Schrepler[5], Mihaela Silion[6], Dana Maniu[1,2], Monica Focsan[2,✉], Simion Astilean[1,2,✉].

[1] Biomolecular Physics Department, Faculty of Physics, Babes-Bolyai University, Cluj-Napoca, Cluj, Romania;
[2] Nanobiophotonics and Laser Microspectroscopy Centre, Interdisciplinary Research Institute on Bio-Nano-Sciences, Babes-Bolyai University, Cluj-Napoca, Cluj, Romania;
[3] The Research Centre on Fundamental and Applied Heterochemistry, Department of Chemistry, Faculty of Chemistry and Chemical Engineering, Babes-Bolyai University, Cluj-Napoca, Cluj, Romania;
[4] Department of Pharmaceutical Chemistry, Faculty of Pharmacy, 'Iuliu Hațieganu' University of Medicine and Pharmacy, 41 Victor Babes¸ Street, Cluj-Napoca 400012, Romania
[5] Department of Radiobiology and Tumor Biology, Oncology Institute Prof. Dr. Ion Chiricuta, Cluj-Napoca, Cluj, Romania;
[6] Romanian Academy of Sciences Petru Poni Institute of Macromolecular Chemistry Romanian Acad, Adv Res Ctr Bionanoconjugates & Biopolymers, Petru Poni Inst Macromol Chem, Iasi, Romania;
✉ Corresponding author

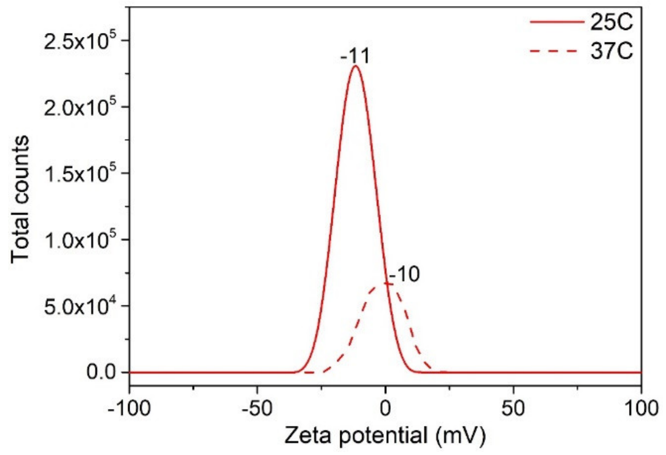

Figure S1. Zeta potential measurements of the BSA&phthaloNH_2 NPs at 25 ºC (solid line) and at 37 ºC (dashed line).

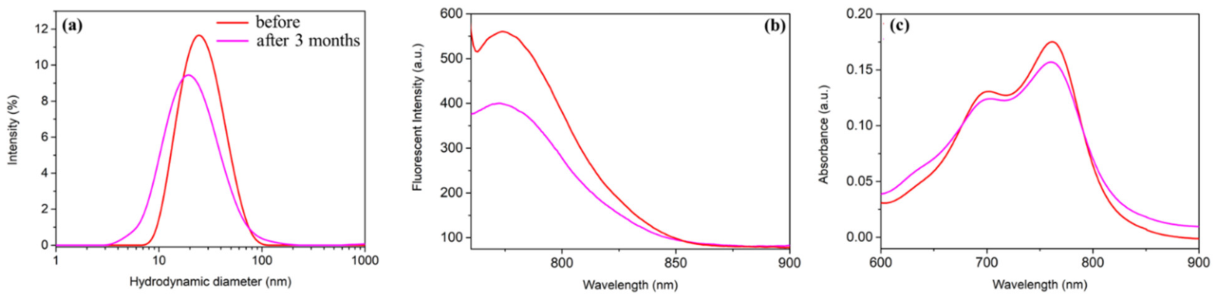

Figure S2. (**a**) DLS, (**b**) fluorescence and (**c**) absorption spectra of the BSA&phthaloNH_2 NPs just after development (red) and after 3 months of storage (pink).

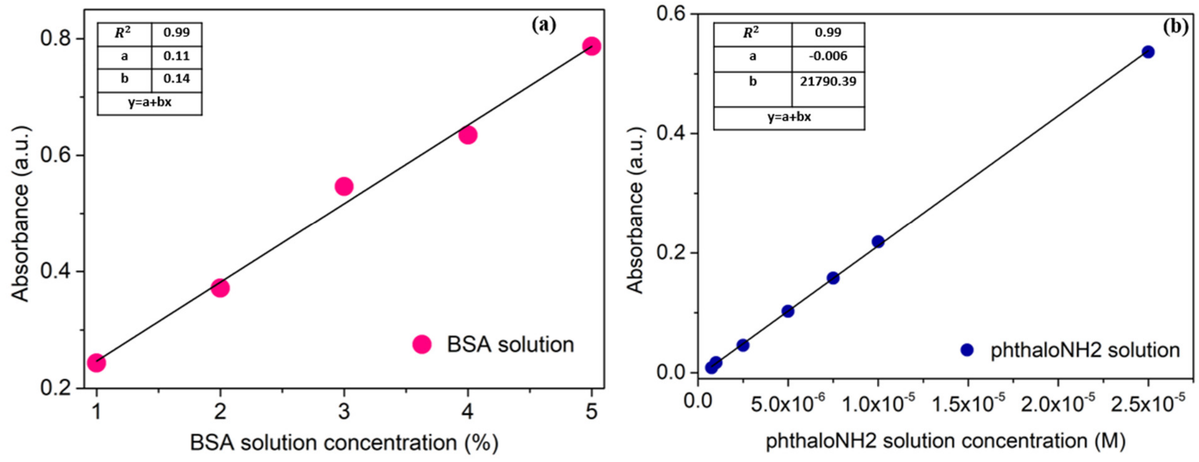

Figure S3. The calibration curves for different concentrations of **(a)** free BSA **(b)** phthaloNH_2 dye solution.

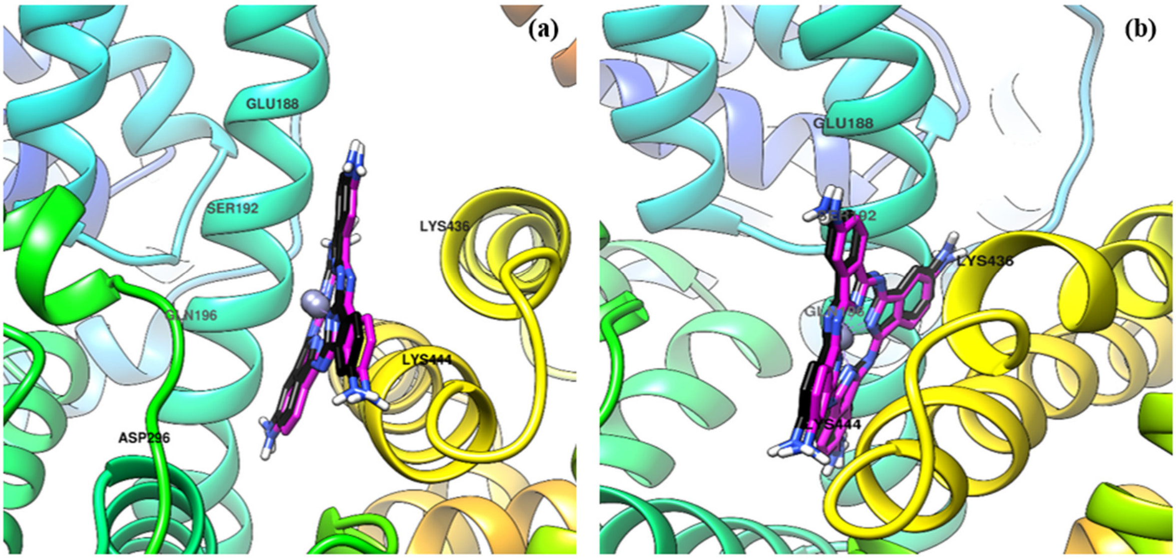

Figure S4. View from both sides of the poses predicted for phthaloNH2 into the two Sudlow's site I by **(a)** AutoDock (carbon atoms in magenta) and **(b)** AutoDock vina (carbon atoms in black). Some amino acid labels were depicted for a better comprehension.

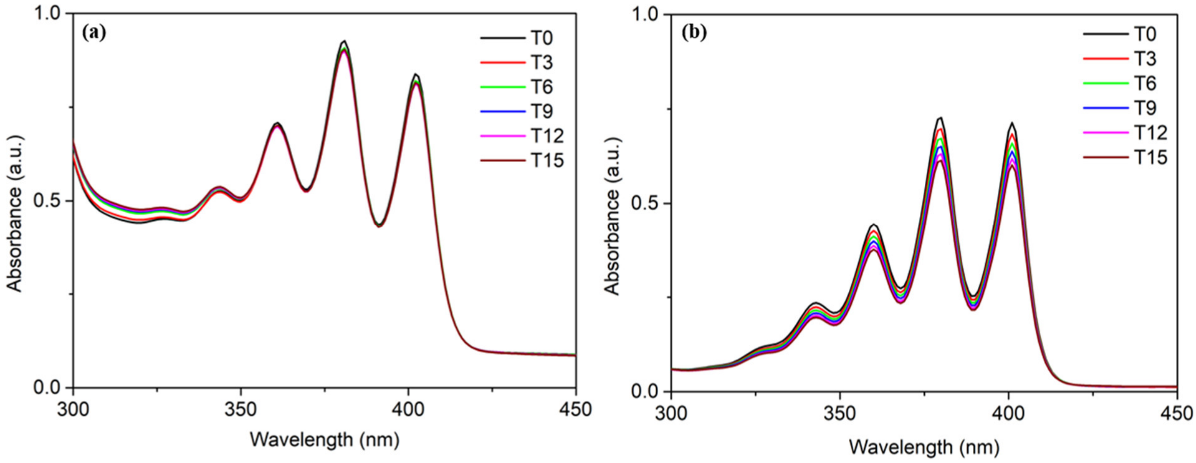

Figure S5. Degradation of ABDA during 15 minutes of irradiation in the presence of **(a)** BSA&phthaloNH2 NPs and **(b)** free phthaloNH2.

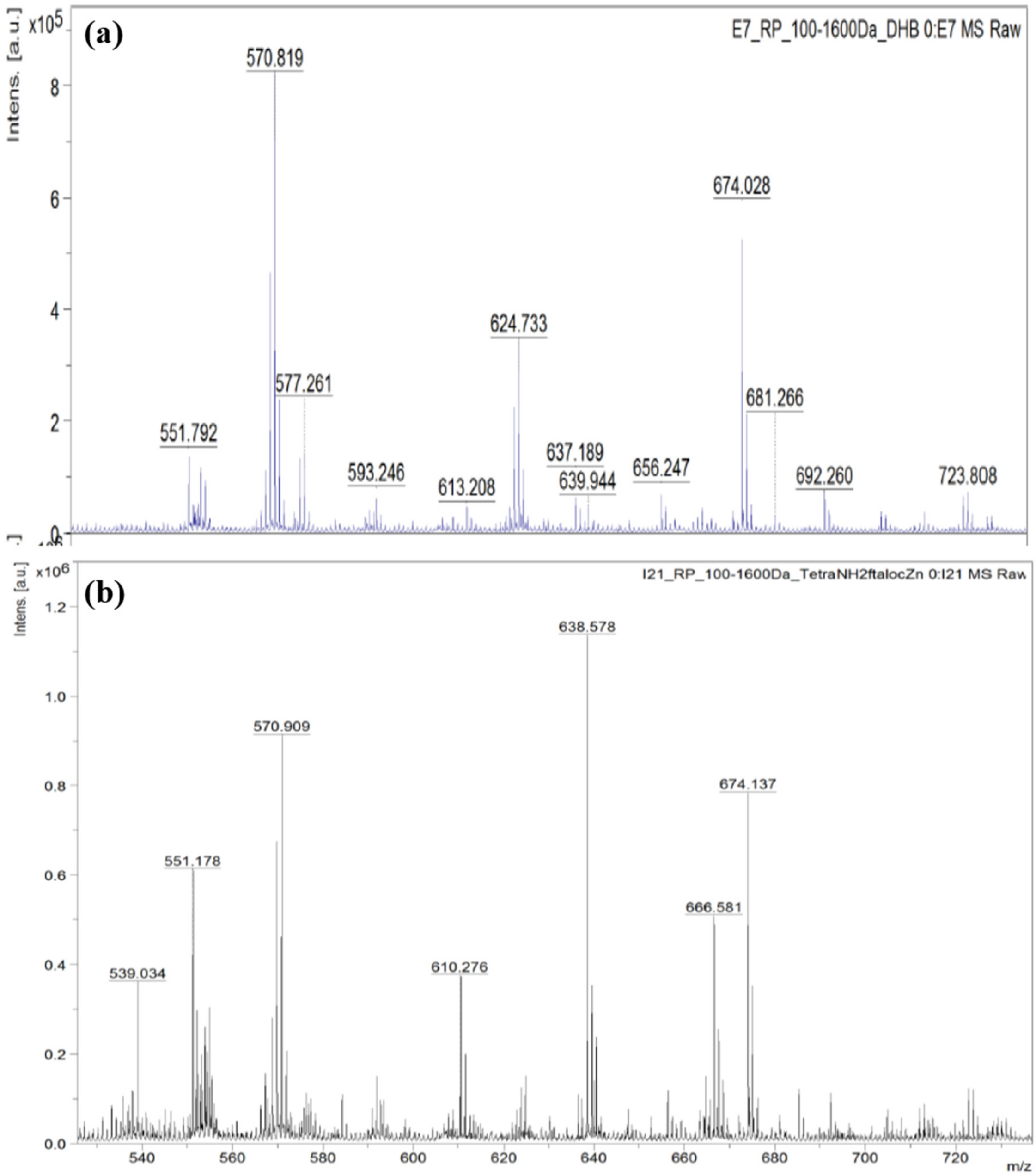

Figure S6. Mass spectra (MALDI-TOF-MS): (**a**) the DHB (2,5-dihydroxybenzoic acid) matrix and (**b**) Zn (II)-2,9,16,23-tetraaminophthalocyanine (m/z = 638.578) in the presence of the DHB matrix.
